# Cadmium stress dictates central carbon flux and alters membrane composition in *Streptococcus pneumoniae*

**DOI:** 10.1038/s42003-020-01417-y

**Published:** 2020-11-19

**Authors:** Stephanie L. Neville, Bart A. Eijkelkamp, Amber Lothian, James C. Paton, Blaine R. Roberts, Jason W. Rosch, Christopher A. McDevitt

**Affiliations:** 1 Department of Microbiology and Immunology, The Peter Doherty Institute for Infection and Immunity, The University of Melbourne, Melbourne, VIC Australia; 2 College of Science and Engineering, Flinders University, Bedford Park, SA Australia; 3 Melbourne Dementia Research Centre, The Florey Institute of Neuroscience and Mental Health, The University of Melbourne, Parkville, VIC Australia; 4 Research Centre for Infectious Diseases, Department of Molecular and Biomedical Science, University of Adelaide, Adelaide, SA Australia; 5 Department of Biochemistry, Emory University School of Medicine, Atlanta, GA 30322 USA; 6 Department of Infectious Diseases, St Jude Children’s Research Hospital, Memphis, TN USA

**Keywords:** Bacteriology, Metals, Metabolic pathways, Pathogens

## Abstract

Metal ion homeostasis is essential for all forms of life. However, the breadth of intracellular impacts that arise upon dysregulation of metal ion homeostasis remain to be elucidated. Here, we used cadmium, a non-physiological metal ion, to investigate how the bacterial pathogen, *Streptococcus pneumoniae*, resists metal ion stress and dyshomeostasis. By combining transcriptomics, metabolomics and metalloproteomics, we reveal that cadmium stress dysregulates numerous essential cellular pathways including central carbon metabolism, lipid membrane biogenesis and homeostasis, and capsule production at the transcriptional and/or functional level. Despite the breadth of cellular pathways susceptible to metal intoxication, we show that *S. pneumoniae* is able to maintain viability by utilizing cellular pathways that are predominately metal-independent, such as the pentose phosphate pathway to maintain energy production. Collectively, this work provides insight into the cellular processes impacted by cadmium and how resistance to metal ion toxicity is achieved in *S. pneumoniae*.

## Abstract (web summary)

Neville et al. investigate how *Streptococcus pneumoniae* mitigates metal ion stress. Despite cadmium induced dysregulation of central carbon metabolism and lipid membrane homeostasis, they find that *S. pneumoniae* can remain viable by selectively utilizing predominately metal-independent cellular pathways. This study provides insights into how bacteria overcome metal ion toxicity.

## Introduction

Cadmium (Cd) is a naturally abundant metal in the Earth’s crust. However, industrialization has dramatically increased its flux into the biosphere, driven by processes including urban waste disposal, phosphate-based fertilizer usage, non-ferrous iron ore processing and battery disposal^1,2^. The ecological impact of anthropogenic Cd release is highlighted by increased accumulation of the metal in topsoils, and contamination of marine and terrestrial ecosystems^3,4^. The flux and accumulation of Cd in these environments has increased its rate of entry into the food chain with food consumption serving as a major route of human Cd intake (10–26 µg/day^3^) with substantial bioaccumulation (0.6–1.3 µg/day^5^). Cadmium is acutely toxic in biological systems, with an estimated biological half-life of 17–30 years^6^. The physiological consequences of Cd exposure are well-documented^7^, but despite this, the molecular basis of toxicity remains to be fully understood^8^. Current models propose that Cd, which occurs as the divalent cation Cd^2+^ in biological systems, exerts toxicity via the generation of reactive oxygen species that mediate DNA damage and lipid peroxidation^9–11^. However, this may be an indirect effect arising from the thiophilicity of the metal ion and its ability to coordinate with nitrogen and oxygen-contributing side chains present in the metal-binding sites of metalloproteins^12^. Cadmium competition for magnesium, calcium, manganese (Mn^2+^) or zinc (Zn^2+^) binding sites could perturb or abrogate metalloprotein function due to the acquisition of a non-cognate metal cofactor^13^. Recent bioinorganic chemical studies have provided support for this inference, with specific examples of Cd^2+^-mismetallation including Zn^2+^-finger DNA-binding proteins, leading to perturbation of DNA repair mechanisms^14,15^, and metalloregulatory proteins, leading to dysregulation of metal ion homeostasis^16–18^. Succinctly, the ability of Cd^2+^ to readily accumulate within cellular systems and inappropriately interact with metalloproteins contributes to its toxicity. Thus, investigation of Cd^2+^ toxicity provides a unique framework to elucidate the cellular impacts associated with a break down in the homeostasis of biologically important metal ions.

Here, we have used Cd^2+^ to investigate how metal intoxication impacts essential cellular processes in *Streptococcus pneumoniae* (the pneumococcus). *S. pneumoniae* is a Gram-positive human pathogen that is exposed to multiple inorganic chemical stresses during infection, such as Mn^2+^-limitation and Cu^2+^- and Zn^2+^-intoxication^19–22^. Although Cd^2+^ is a non-physiological metal ion, recent studies have indicated Cd^2+^ stress may also occur during colonization and infection of the lungs of tobacco smokers. Cigarettes contain high levels of Cd^2+^ (0.5–1 μg per cigarette^23^) due to the Cd^2+^ hyper-accumulative properties of *Nicotiana tabacum* leaves. Aerosolization of Cd^2+^ facilitates more rapid absorption via the lungs than through food consumption^24^ with recent studies showing persistent bioaccumulation of Cd^2+^ in lung tissues and bronchoalveolar lavage fluids^25^ in tobacco smokers. Moreover, *S. pneumoniae* exposed to tobacco smoke manifests a transcriptional response consistent with Cd^2+^ exposure^26^. Although questions remain regarding the effect of inorganic chemical insults during pneumococcal infection, the identity and importance of the metal homeostasis mechanisms are well-defined^20,22,27–30^. Prior work from our group has shown that *S. pneumoniae* acquires Cd^2+^ via the Mn^2+^-specific ABC permease, PsaBCA, and rapidly accumulates in the cytoplasm due to the absence of an efflux system^18^. In addition, *S. pneumoniae* has a comparatively limited metabolic capacity compared to many other bacterial pathogens, lacking an electron transport chain, a complete tricarboxylic acid cycle and an Entner-Dourdoroff pathway. These factors, in combination with the single cellular compartment of the organism and the lack of any native Cd^2+^ utilization by *S. pneumoniae*, provide a simplified system in which to dissect the complex molecular impacts of dysregulated metal ion homeostasis.

We used an integrated multi-omics approach that combined transcriptomics, metabolomics, and metalloproteomics to address this question. This revealed the global effects of Cd^2+^-mediated dysregulation of metal homeostasis on gene expression, metabolism, and metalloprotein metallation status. Essential cellular processes that were perturbed included inorganic ion (Mn^2+^ and Zn^2+^) homeostasis, corroborating our previous findings^18^, but also carbon metabolism and fatty acid (FA) biosynthesis. Notably, Cd^2+^-intoxication exerted a profound inhibitory effect on glycolysis, mediated by reduced gene expression and impaired protein function. Glucose catabolism proceeded via the pentose phosphate pathway (PPP), circumventing the impaired glycolytic enzymes, and resulting in a shift to mixed acid fermentation. Intoxication with Cd^2+^ also impacted FA biosynthesis and manifested as an alteration in membrane phospholipid composition and reduced membrane fluidity. The shift in glucose flux and upregulation of alternative, metal-independent enzymes, such as GapN, during exposure to Cd^2+^-intoxication suggest the presence of alternate metabolic pathways that facilitate survival during exposure to conditions that disrupt cellular metal homeostasis. Collectively, this study provides new insights into the molecular basis of Cd^2+^ toxicity, the breadth of impacts that arise from disruption of cellular metal ion homeostasis, and reveals the mitigation strategies that bacteria, such as *S. pneumoniae*, can employ to survive these chemical stress insults.

## Results

### Cadmium stress impacts *S. pneumoniae* growth and exerts global transcriptome changes

**Fig. 1:**
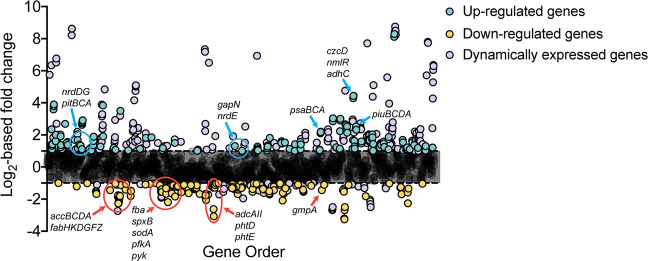
*S. pneumoniae* transcriptome in response to 30 µM Cd^2+^ stress. Gene expression profile of mid-log phase *S. pneumoniae* D39 grown in the presence of Cd^2+^ stress (30 μM Cd^2+^) compared to untreated. Genes are plotted along the *x*-axis, ordered according to locus tag (1–2069). Differential expression of genes in response to Cd^2+^ stress is displayed in Log_2_ values along the *y*-axis. Genes upregulated >2-fold have been colored blue. Genes downregulated by >2-fold have been colored yellow. Dynamically expressed genes as defined by Aprianto et al.^46^ have been colored purple. Annotated genes have been discussed further.

Exposure to 30 μM Cd^2+^ perturbs pneumococcal growth resulting in an extended lag time (0.11 vs. 0.19 h) and a reduction in the maximal growth rate (1.10 vs. 0.66 h^−1^) (Supplementary Fig. 1a, Supplementary Table 1). This effect has previously been ascribed to Cd^2+^-induced perturbation of cellular accumulation of manganese (Mn^2+^) and zinc (Zn^2+^) ions^18^, but the extent of the cellular impact of Cd^2+^ was not fully defined. Accordingly, we investigated the global transcriptional response of *S. pneumoniae* to 30 μM Cd^2+^ stress. Here, we observed that exposure to 30 μM Cd^2+^ resulted in transcriptional changes of >2-fold for 544 (26%) genes and >4-fold to 152 (7%) genes (Fig. 1) relative to untreated cells. Of the genes that showed differences of more than two-fold, 357 (17%) were upregulated, and 187 (9%) were downregulated. Validation of the RNA sequencing data were performed by qRT-PCR analysis using a subset of differentially expressed genes (Supplementary Fig. 2, Supplementary Table 2).

The largest proportion of significantly (>2-fold) upregulated genes belonged to the Clusters of Orthologous Groups (COG) defined categories of carbohydrate metabolism (30.1%) and inorganic ion homeostasis (24.4%), while genes associated with lipid metabolism represented the largest downregulated group (29.7%) (Supplementary Fig. 3). Genes upregulated to the greatest extent were associated with pneumococcal competence, within both the early (ComE) and late (ComX) competence regulons, and bacteriocin production (BlpR regulon) (Table 1)^31^. Genes comprising the pneumococcal competence and bacteriocin production regulons have highly dynamic transcriptional profiles and are readily induced by a variety of environmental factors including pH, oxygen tension, chemical and nutrient stress^28,32–36^. Our data show that Cd^2+^ stress also activates these regulons, which can likely be attributed to the depletion of cellular Mn^2+^ and Zn^2+^, consistent with our prior report^18^, although a direct response to Cd^2+^ cannot be excluded.

**Table 1:** Summary of genes highly upregulated in *S. pneumoniae* upon exposure to 30 μM Cd^2+^.

Locus Tag	Gene/Locus	Predicted function	Log_2_-fold change^a^	Regulon
SPD_1860	*comGD*	Competence protein	+8.75	ComX
SPD_0133	*cibA*	Class IIb bacteriocin	+8.62	ComX
SPD_1862	*comGB*	Competence protein	+8.50	ComX
SPD_1859	*comGE*	Putative membrane protein	+8.46	ComX
SPD_1861	*comGC*	Competence protein	+8.38	ComX
SPD_1857	*comGG*	Uncharacterized protein	+8.37	ComX
SPD_1858	*comGF*	Competence protein	+8.36	ComX
SPD_1863	*comGA*	Competence protein	+8.23	ComX
SPD_0132	*cibB*	Putative bacteriocin	+8.21	ComX
SPD_2034	*comFC*	Competence protein	+7.80	ComX
SPD_2035	*comFA*	Competence protein	+7.74	ComX
SPD_1711	*ssbB*	Single-strand DNA-binding protein	+7.71	ComX
SPD_0843	*comEA*	Competence protein	+7.34	ComX
SPD_0844	*comEC*	Competence protein	+7.18	ComX
SPD_1122	*dprA*	DNA processing protein	+6.93	ComX
SPD_0050	*comB*	Competence factor transport protein	+6.76	ComE
SPD_0049	*comA*	Competence factor transporting protein	+6.51	ComE
SPD_0865	*coiA*	Competence protein	+6.50	ComX
SPD_2064	*comD*	Sensor histidine kinase	+6.36	RpoD, ComE
SPD_0023	*comW*	Uncharacterized protein	+6.34	ComE
SPD_2065	*comC1*	Competence-stimulating peptide type 1	+6.29	RpoD, ComE
SPD_2063	*comE*	Response regulator	+6.05	RpoD, ComE
SPD_0014	*comX1*	Transcriptional regulator	+5.75	ComE
SPD_2028	*cbpD*	Choline binding protein D	+5.71	ComX
SPD_1818	*comX2*	Transcriptional regulator	+5.59	ComE
SPD_1744	*comM*	Competence self-immunity protein	+5.10	ComE
SPD_0475	*pncP*	Bacteriocin self-immunity protein	+5.09	ComE, BlpR
SPD_0474	*blpZ*	Uncharacterized protein	+5.02	ComE, BlpR
SPD_0308	*clpL*	ATP-dependent Clp protease	+4.84	CtsR, RpoD
SPD_1593	*cclA*	Type IV prepilin peptidase	+4.76	ComX
SPD_0473	*blpY*	Bacteriocin self-immunity protein	+4.74	ComE, BlpR
SPD_1638	*czcD*	Cation diffusion facilitator protein	+4.44	SczA
SPD_1637	*nmlR*	MerR family transcriptional regulator	+4.43	NmlR, SczA
SPD_1740	*cinA*	Competence-damage inducible protein	+4.38	ComX
SPD_1636	*adhC*	Zn^2+^-containing alcohol dehydrogenase	+4.29	NmlR, SczA
SPD_0466	*blpT*	Uncharacterized bacteriocin protein	+4.16	ComE, BlpR

^a^Log_2_-fold change in gene expression comparing *S. pneumoniae* D39 in cation-defined media (CDM) relative to CDM supplemented with 30 μM Cd^2+^.

The next most highly upregulated group of genes were *czcD*, *nmlR,* and *adhC*, which comprise the overlapping SczA/NmlR regulons and belong to the inorganic ion homeostasis COG (Table 1). SczA is a cation-dependent, TetR family transcriptional regulator, which is primarily activated by Zn^2+^ in *S. pneumoniae*, but can also be regulated by other metals including cobalt, nickel and Cd^2+^ ^18,37^. Transcriptional activation by SczA leads to the expression of *czcD*, a cation diffusion facilitator pathway that can export Zn^2+^ and Co^2+^ ions from *S. pneumoniae*. However, CzcD does not export Cd^2+^ and, as a consequence, induction of *czcD* by Cd^2+^-SczA results in excessive Zn^2+^ efflux. Thus, our transcriptional observations are concordant with Zn^2+^ accumulation studies of Cd^2+^-intoxicated *S. pneumoniae*^18^. NmlR is a MerR family transcriptional regulator that controls expression of the Zn^2+^-dependent class III alcohol dehydrogenase, AdhC. Although the precise functional roles of NmlR and AdhC remain poorly defined, NmlR has been suggested to be an aldehyde-responsive regulator, while AdhC has been proposed to be involved in a variety of roles including carbohydrate metabolism, detoxification of reactive aldehydes and dicarbonyl compounds produced from triose sugars during carbon metabolism, and/or regenerating glutathione from glutathione-aldehyde adducts^38,39^. In addition, other genes involved in inorganic ion homeostasis also showed altered expression (Supplementary Table 3). Consistent with our earlier studies^18^, the genes involved in Mn^2+^ uptake were upregulated. Intriguingly, although Cd^2+^-stress did not impact cellular iron abundance, we observed upregulation of two iron import gene clusters, the *pitCBDA* and *piuBCDA* permeases, while the genes associated with a putative hemin importer, *SPD_1588-1591*, and *piaB*, a component of the Fe-hydroxamate import pathway, were downregulated. These changes in iron acquisition pathway expression can most likely be attributed to the metalloregulatory protein RitR responding to the reduced Mn^2+^ abundance during exposure to Cd^2+^-stress^40,41^.

In summary, the transcriptomics showed that cellular Cd^2+^ accumulation results in broad transcriptional changes in *S. pneumoniae* and consistent with our previous findings^18^, impacts pathways that employ metalloproteins and/or are associated with regulation of cellular metal homeostasis. Thus, we sought to elucidate how Cd^2+^ stress influenced metal-cofactor abundance in the pneumococcus.

### Cadmium stress impacts the *S. pneumoniae* metalloproteome

Zinc and Mn^2+^ are the most abundant first-row transition metal ions in *S. pneumoniae*^18,42^. It therefore follows that Cd^2+^-mediated dysregulation of Zn^2+^ and Mn^2+^ accumulation would have substantial effects on metalloproteins and metal-cofactor abundance. Here, we investigated the impact of Cd^2+^ accumulation by combining liquid chromatography-inductively coupled plasma-mass spectrometry (LC-ICP-MS) with protein mass spectrometry (MS) to generate proteomic maps of the cellular distribution of metal ions, i.e., the metalloproteome. The metalloproteome was visualized by two-dimensional separation of the proteome with metal ions detected by ICP-MS and represented as metal concentration in parts per billion (ppb). The two-dimensional separation of the proteome was performed by anion exchange (AEX) and size exclusion chromatography (SEC), which provided chromatographic distribution by resolving proteins of differing charge and mass, respectively. At equivalent total protein, variation in the concentration of metal (in ppb) and distribution of metal peaks can then be compared between Cd^2+^-treated and untreated samples to determine the impact of Cd^2+^ stress and its cellular accumulation on metalloprotein cofactor abundance.

**Fig. 2:**
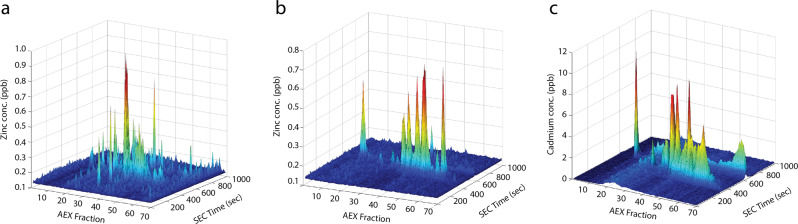
Metalloproteomic maps of untreated and Cd^2+^-treated *S. pneumoniae*. Detection and distribution of Zn^2+^- and Cd^2+^-associated proteins throughout the pneumococcal metalloproteome. **a** Zn^2+^-associated proteins in untreated *S. pneumoniae*, **b** Zn^2+^-associated proteins in Cd^2+^-treated *S. pneumoniae*, **c** Cd^2+^-associated proteins in Cd^2+^-treated *S. pneumoniae*. Anion exchange (AEX) separation of proteins is shown on the *x*-axis and size exclusion chromatography (SEC) separation of proteins (time in seconds) is shown on the *y* axis. The concentration of metal detected (in parts per billion [ppb]) is shown on the *z*-axis. All metalloproteomic maps are representative of 2 mg total cytoplasmic protein.

Using this framework, the distribution of Zn^2+^ in the pneumococcal proteome was assessed (Fig. 2a) and contrasted with Zn^2+^ distribution in Cd^2+^-treated *S. pneumoniae* (Fig. 2b). Metalloproteomic maps of the cytoplasmic proteome show that protein-associated Zn^2+^ was directly affected with the number of protein-Zn^2+^ interactions (denoted by peaks) decreasing ~30% in Cd^2+^-treated cells compared to untreated (428 peaks, untreated vs. 306 peaks, Cd^2+^-treated; Fig. 2a, b). The decreased quantity and spatial distribution of Zn^2+^ peaks (protein-associated Zn^2+^) in the Cd^2+^-treated map suggests that there are fewer protein-Zn^2+^ interactions across a comparatively limited subset of cytoplasmic proteins during Cd^2+^ stress. This may be due to the overall reduction in cellular Zn^2+^ that occurs during Cd^2+^ stress^18^, Cd^2+^ competition for Zn^2+^ binding sites^12^, or a combination of these processes. To address this, the distribution of Cd^2+^-associated proteins in the treated pneumococcal proteome was assessed (Fig. 2c). Concordant with Cd^2+^-treatment, the pneumococcal proteome revealed 395 individual peaks, indicative of protein-Cd^2+^ interactions, and comparatively higher Cd^2+^ abundance across the cytoplasmic proteome (Supplementary Fig. 4). Comparison of the Cd^2+^ peaks with the Zn^2+^ proteome showed overlapping spatial distribution of the peaks, suggesting that Cd^2+^ ions were interacting with some of the fractions previously associated with Zn^2+^ ions (Supplementary Fig. 4). The distribution of Mn^2+^ ions in the metalloproteome was also investigated. However, due to the extent of Mn^2+^-depletion in Cd^2+^-treated cells^18^, robust maps could not be generated for meaningful comparisons. We then sought to ascertain the identities of the proteins putatively mismetallated by Cd^2+^ ions.

*Streptococcus pneumoniae* does not contain Cd^2+^-dependent proteins. Thus, it logically follows that protein association with Cd^2+^ is indicative of mismetallation. Mass spectrometry performed on the fractionated cytoplasmic proteome revealed the identities of many cellular proteins (Supplementary Data 1), which were data-mined for both characterized (Uniprot, BRENDA) and uncharacterized metalloproteins (NCBI Conserved Domains). The definitively identified metalloproteins (score ≥ 24) contained within specific Cd^2+^-enriched peaks are detailed in Table 2. The major cellular processes with apparent Cd^2+^-mismetallation impacts were carbohydrate metabolism, inorganic ion homeostasis and nucleotide biosynthesis. For inorganic ion homeostasis, the proteins identified within Cd^2+^-enriched peaks were the Mn^2+^-recruiting protein PsaA and the Zn^2+^-dependent transcriptional regulator AdcR. Previously, we have shown that PsaA can bind Cd^2+^ with high affinity and contributes to its cellular import^18^. Thus, our metalloproteomics data support our prior conclusions and highlight the utility of this technique to identify metalloproteins which may be susceptible to Cd^2+^ mismetallation. These data also provide additional insight into the molecular basis of Cd^2+^-mediated dysregulation of Zn^2+^ homeostasis. AdcR is a MarR family regulator that binds to DNA in the Zn^2+^-bound state, negatively regulating gene expression. Under conditions of Zn^2+^-depletion AdcR dissociates from DNA thereby permitting upregulation of the *adc* regulon, which encodes genes involved in Zn^2+^ uptake^27,43,44^. In our previous work, we reported that AdcR function was dysregulated during Cd^2+^ stress, potentially arising from Zn^2+^ being displaced from the thiol buffering pool onto AdcR or by Cd^2+^ mismetallation of AdcR^18^. Here, we report that AdcR was identified within a Cd^2+^ peak (Table 2) providing support for the formation of a mismetallated Cd^2+^-bound AdcR complex. We propose that the resultant depletion of cellular Zn^2+^ can be attributed to Cd^2+^-bound AdcR mimicking the Zn^2+^-bound state leading to repression of the *adc* regulon consistent with the downregulation of the Zn^2+^-recruiting genes *adcAII*, *phtB*, *phtD,* and *phtE*, (Supplementary Table 3). Taken together, the transcriptomic and metalloproteomic data provide insights into the mechanistic basis for Cd^2+^-mediated dysregulation of Zn^2+^ homeostasis.

**Table 2:** Metalloproteins identified in Cd^2+^-enriched peaks.

Locus tag	Protein	Accession number	Predicted function	Metal cofactor	Score	Number of peptides found	Sequence coverage (%)	Containing Fraction(s)
SPD_0577	ZmpB	ZMPB_STRR6	Zinc metalloprotease (EC 3.4.24.-)	Zn^2+^	25	61	27	11
SPD_0789	PfkA	PFKA_STRPS	Phosphofructokinase (EC 2.7.1.11)	Mg^2+^	39	13	29	32,33
SPD_2000	AdcR	ADCR_STRP2	MarR family transcriptional repressor	Zn^2+^	40	6	14	34
SPD_0526	Fba	ALF_STRPN	Fructose-1,6-bisphosphate aldolase (EC 4.1.2.13)	Zn^2+^	430	57	63	32,33,34
SPD_0636	SpxB	POXB_STRPN	Pyruvate oxidase (EC 1.2.3.3)	Mg^2+^	111	24	29	32,34,37
SPD_1390	GlmM	GLMM_STRP4	Phosphoglucosamine mutase (EC 5.4.2.10)	Mg^2+^	137	23	32	47,49,51,53
SPD_0309	LuxS	LUXS_STRPI	S-ribosylhomocysteine lyase	Fe^3+^	102	15	51	47,49,51
SPD_1463	PsaA	MTSA_STRPN	Mn^2+^ ABC transporter substrate-binding protein	Mn^2+^	27	8	25	47,49,51
SPD_0874	GlmU	GLMU_STRP4	UDP-N-acetylglucosamine pyrophosphorylase	Mg^2+^	100	87	46	49,51,53,55
SPD_1012	Eno	ENO_STRPI	Enolase (EC 4.2.1.11)	Mg^2+^	152	28	32	55,57,59
SPD_0389	AccD	ACCD_STRPN	Acetyl-CoA carboxylase subunit D (EC 6.4.1.2)	Zn^2+^	24	18	32	59
SPD_0012	Hpt	A0A0H2ZP27	Hypoxanthine-guanine phosphoribosyltransferase (EC:2.4.2.8)	Mg^2+^	38	6	34	57
SPD_0024	PurA	PURA_STRP2	Adenylosuccinate synthetase (EC:6.3.4.4)	Mg^2+^	30	20	43	32,33,34
SPD_1839	Tkt/UlaH	A0A0H2ZLU9	Transketolase (EC:2.2.1.1)	Mg^2+^	33	21	32	37,38,41
SPD_0724	DeoB	DEOB_STRP2	Phosphopentomutase (EC:5.4.2.7)	Mn^2+^	59	9	19	38,41,47
SPD_1484	Ddl	DDL_STRP2	D-alanine--D-alanine ligase (EC:6.3.2.4)	Mg^2+^/Mn^2+^	55	18	48	38
SPD_0667	SodA	A0A0H2ZLU4	Superoxide dismutase (EC:1.15.1.1)	Fe^2+^/Mn^2+^	128	14	62	41
SPD_1363	PpaC	PPAC_STRP2	Probable manganese-dependent inorganic pyrophosphatase (EC:3.6.1.1)	Mn^2+^	330	26	47	49,51
SPD_0894	PepT	PEPT_STRP2	Peptidase T (EC:3.4.11.4)	Zn^2+^	40	9	11	49,51,53
SPD_1434	YgbL	GCH1L_STRPN	GTP cyclohydrolase 1 type 2 homolog	Mg^2+^	28	6	19	57
SPD_0013	FtsH	A0A0H2ZMA2	ATP-dependent zinc metalloprotease FtsH (EC:3.4.24.-)	Zn^2+^	25	12	15	26

### Glucose catabolism is disrupted by cellular Cd^2+^ accumulation

Building on the transcriptional and metalloproteomic profiling, we investigated the impact of Cd^2+^ intoxication on carbohydrate metabolism and FA biosynthesis by metabolomics. *S. pneumoniae* is a fermentative organism that generates energy via substrate-level phosphorylation since it lacks an electron transport chain. Similar to many other lactic acid bacteria *S. pneumoniae* uses glycolysis (Embden-Meyerhof pathway) to catabolize glucose^45^. Transcriptomics revealed that Cd^2+^ stress was associated with a significant downregulation (>2-fold) in the expression of numerous genes involved in glycolysis and the primary glucose PTS importer, *manLMN* (Table 3). Of particular note was the downregulation of the sugar phosphotransferase genes *ptsH* and *ptsI*, and the metabolic genes fructose-1,6-bisphosphate aldolase (*fba*), enolase (*eno*) and pyruvate oxidase (*spxB*). These genes are amongst the most highly expressed in the pneumococcus and are refractory to transcriptional regulation in most environmental conditions^46^. PtsHI has an essential role in mediating phosphoryl transfer from phosphoenolpyruvate (PEP) to the phosphotransferase system (PTS) sugar importers. Thus, downregulation of *ptsHI* would be predicted to broadly impact PTS-mediated carbohydrate import and may be responsible for the reduced expression of the glucose PTS importer *manLMN*. Downregulation of *ptsH* may also indirectly influence the coordination of carbon metabolism due to its interaction with carbon catabolite repression^47^. Downregulation of the highly expressed glycolytic enzymes, *fba*, *eno* and *spxB* would also be expected to alter glycolytic flux and thereby impact energy generation. Notably, the primary carbon catabolite repressor (*ccpA*) was not differentially expressed in response to Cd^2+^, and the differential expression of the aforementioned metabolic genes appears inconsistent with carbon catabolite control via CcpA^48^. This suggests that the altered metabolic response of *S. pneumoniae* to Cd^2+^ is not CcpA-coordinated.

**Table 3:** Differential expression of carbon catabolism and fatty acid biosynthesis genes associated with 30 μM Cd^2+^ stress.

Locus tag	Gene/Locus	Predicted function	Log_2_-fold change^a^
**Increased relative expression**			
Glucose catabolism/glycolysis			
SPD_1004	*gapN*	Glyceraldehyde-3-phosphate dehydrogenase, NADP-dependent (EC 1.2.1.9)	+1.31
Pentose phosphate pathway			
SPD_0289	*eda*	2-deydro-3-deoxyphosphogluconate aldolase (EC 4.1.2.14/4.1.3.16)	+1.82
SPD_0290	SPD_0290	Carbohydrate kinase, PfkB family protein	+1.60
SPD_0291	SPD_0291	Ribose 5-phosphate isomerase, putative	+1.80
Leloir pathway			
SPD_1633	*galT-2*	Galactose-1-phosphate uridylyltransferase (EC 2.7.7.12)	+2.96
SPD_1634	*galK*	Galactokinase (EC 2.7.1.6)	+2.85
SPD_1635	*galR*	Galactose operon repressor	+1.68
Pyruvate metabolism			
SPD_0235	*pfl*	Putative pyruvate formate lyase	+1.86
SPD_1636	*adhC*	Zn^2+^-containing alcohol dehydrogenase (EC:1.1.1.1)	+4.29
**Decreased relative expression**			
Glucose catabolism/glycolysis			
SPD_0262	*manN*	PTS-system, IID component	−1.50
SPD_0263	*manM*	PTS-system, IIC component	−1.59
SPD_0264	*manL*	PTS-system, IIAB components	−1.68
SPD_0526	*fba*	Fructose-1,6-bisphosphate aldolase, class II (EC 4.1.2.13)	−1.09
SPD_0789	*pfkA*	Phosphofructokinase (EC 2.7.1.11)	−1.39
SPD_0790	*pyk*	Pyruvate kinase (EC 2.7.1.40)	−1.31
SPD_1012	*eno*	Enolase (EC 4.2.1.11)	−1.22
SPD_1039	*ptsI*	Phosphocarrier protein HPr	−1.30
SPD_1040	*ptsH*	Phosphoenolpyruvate-protein phosphotransferase (EC 2.7.3.9)	−1.31
SPD_1468	*gpmA*	2,3-bisphosphoglycerate-dependent phosphoglycerate mutase (EC 5.4.2.11)	−1.44
Pyruvate metabolism			
SPD_0420	*pflB*	Formate acetyltransferase (EC 2.3.1.54)	−1.41
SPD_0636	*spxB*	Pyruvate oxidase (EC 1.2.3.3)	−1.33
Fatty acid biosynthesis			
SPD_0378	*fabM*	Enoyl-CoA hydratase/isomerase family protein (EC:5.3.3.14)	−2.74
SPD_0379	*fabT*	Transcriptional repressor	−1.86
SPD_0380	*fabH*	3-oxoacyl-[acyl-carrier-protein] synthase 3 (EC:2.3.1.180)	−1.76
SPD_0381	*acaP*	Acyl carrier protein	−2.27
SPD_0382	*fabK*	Trans-2-enoyl-ACP reductase II (EC:1.3.1.9)	−2.27
SPD_0383	*fabD*	Malonyl CoA-acyl carrier protein transacylase (EC:2.3.1.39)	−2.18
SPD_0384	*fabG*	3-oxoacyl-[acyl-carrier-protein] reductase (EC:1.1.1.100)	−2.19
SPD_0385	*fabF*	3-oxoacyl-[acyl-carrier-protein] synthase 2 (EC:2.3.1.179)	−2.04
SPD_0386	*accB*	Acetyl-CoA carboxylase, biotin carboxyl carrier protein	−1.99
SPD_0387	*fabZ*	3-hydroxyacyl-[acyl-carrier-protein] dehydratase (EC:4.2.1.59)	−1.92
SPD_0388	*accC*	Acetyl-CoA carboxylase, biotin carboxylase (EC:6.4.1.2 6.3.4.14)	−1.86
SPD_0389	*accD*	Acetyl-CoA carboxylase carboxyl transferase subunit β (EC:6.4.1.2 2.1.3.15)	−1.79
SPD_0390	*accA*	Acetyl-CoA carboxylase carboxyl transferase subunit α (EC:6.4.1.2 2.1.3.15)	−1.81
SPD_0684	*bioY*	Biotin transporter, putative	−1.84

^a^Log_2_-fold change in gene expression comparing *S. pneumoniae* D39 in CDM supplemented with 30 μM Cd^2+^ relative to CDM alone.

**Fig. 3:**
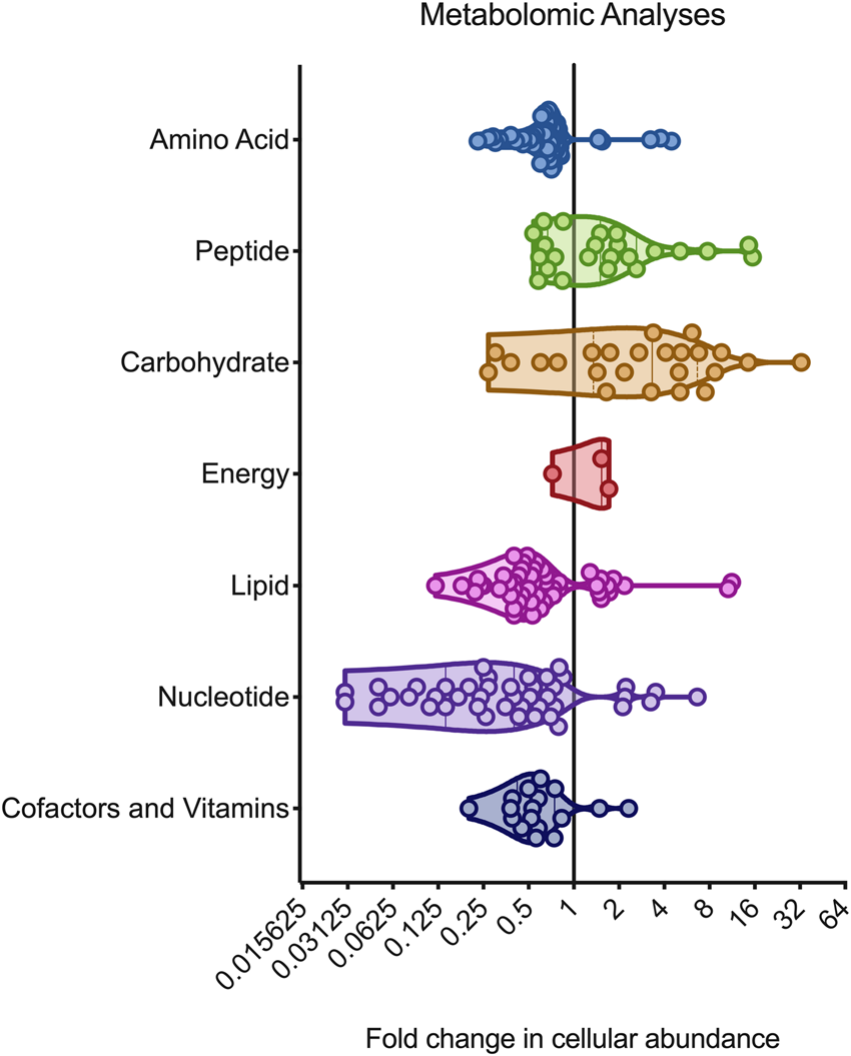
Cadmium induced changes to the *S. pneumoniae* metabolome. Changes in metabolite abundance in *S. pneumoniae* grown in the presence of 30 μM Cd^2+^ stress compared to untreated. Metabolites have been grouped by broad metabolic pathway with fold-change in cellular abundance denoted along the *x*-axis. Each spot represents an individual metabolite, with the shaded areas illustrating violin plots of the frequency distribution of the data. Data presented are the mean fold change in abundance of individual metabolites with statistically significant changes in abundance across six independent biological replicates (*n* = 6). Metabolite listings are provided in Supplementary Data 2.

**Fig. 4:**
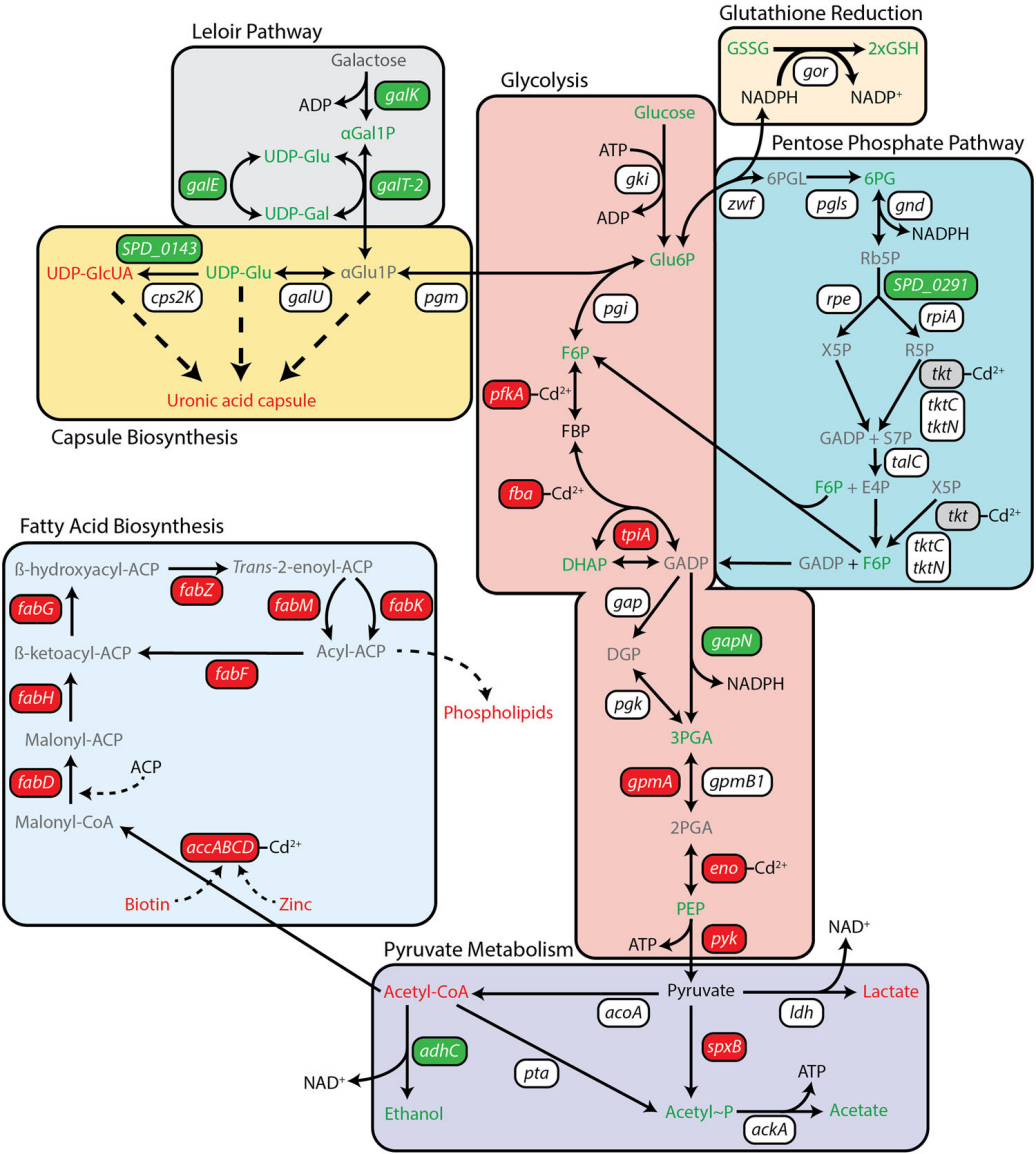
Overview of Cd^2+^-induced impacts to central carbon metabolism in *S. pneumoniae*. Cartoon representation of metabolic pathways associated with central carbon metabolism, capsule biosynthesis and fatty acid biosynthesis. Pathways have been labeled and shaded accordingly. Genes are presented in boxes, with transcriptionally upregulated (>2-fold) genes shown in green boxes, transcriptionally downregulated (>2-fold) genes in red boxes, and genes with changes of <2-fold in white boxes. Metabolomic data are presented as the differential coloring of metabolites (text only) found in the corresponding metabolic pathways. Metabolites in green were accumulated to a significantly higher abundance in Cd^2+^-stressed cells, compared to untreated (*P* < 0.05). Metabolites in red were significantly depleted in abundance in Cd^2+^-stressed cells, compared to untreated (*P* < 0.05). Metabolites in black were not significantly different from untreated cells. Metabolites in gray were not detected in the metabolomic analysis. Metalloproteomic identification of proteins associated within a Cd^2+^ peak, have been annotated with ‘Cd^2+^’ next to the corresponding gene. Gene, protein, and metabolite names can be found on Tables 2, 3 and 4.

Glucose catabolism also relies upon the enzymatic activity of numerous metalloproteins. Here, we identified the Mg^2+^-dependent metalloenzymes phosphofructokinase A (PfkA) and enolase (Eno), and the Zn^2+^-dependent fructose-1,6-bisphosphate aldolase (Fba) in Cd^2+^-enriched peaks of the metalloproteomics indicating possible mismetallation. To address whether the cellular accumulation of Cd^2+^ affected glucose catabolism, we performed metabolomic analyses on *S. pneumoniae* exposed to 30 μM Cd^2+^. Metabolomics detected 416 metabolites and revealed that 162 (39%) were significantly less abundant and 63 (15%) significantly more abundant in Cd^2+^-treated cells, by comparison with untreated cells (*P* < 0.05) (Fig. 3). With respect to glucose catabolism, we observed an increase in the glycolytic precursors glucose, glucose-6-phosphate (Glu6P) and fructose 6-phosphate (F6P), indicating that flux into glycolysis was reduced or impaired prior to the formation of fructose 1,6 bisphosphate (FBP) in Cd^2+^-treated cells (Fig. 4 and Table 4). This finding is consistent with Cd^2+^-mismetallation of PfkA and/or Fba resulting in perturbation or abrogation of their cellular function. Despite the impaired flux into glycolysis, the pneumococcus could circumvent the blockage as the abundance of products from the latter stages of glycolysis, i.e., dihydroxyacetone phosphate (DHAP), 3-phosphoglycerate (3PGA) and PEP, were all observed to increase in abundance.

**Table 4:** Differential accumulation of cellular metabolites associated with central carbon metabolism.

Metabolite	Full name	Fold change in abundance^a^
**Glucose catabolism/glycolysis**		
Glu	Glucose	6.64
Glu6P	Glucose-6-phosphate	8.69
F6P	Fructose-6-phosphate	6.79
FBP	Fructose 1,6-bisphosphate	0.89
DHAP	Dihydroxyacetone phosphate	1.32
3PGA	3-phosphoglycerate	1.43
PEP	Phosphoenolpyruvate	5.02
**Pentose phosphate pathway**		
6PG	6-phosphogluconate	9.59
F6P	Fructose-6-phosphate	6.79
**Pyruvate metabolism**		
Pyruvate	Pyruvate	1.68^b^
Acetyl-P	Acetyl-phosphate	1.71
Acetyl-CoA	Acetyl-CoA	0.23
Ethanol	Ethanol	2.5
Lactate	Lactate	0.27
Acetate	Acetate	1.1
**Glutathione recycling**		
GSH	Glutathione (reduced)	4.47
GSSG	Glutathione (oxidized)	1.46
**Leloir pathway/capsule biosynthesis**		
αGal1P	α-galactose-1-phosphate	5.09
UDP-Glu	UDP-glucose	3.27
UDP-Gal	UDP-galactose	2.71
UDP-GlcUA	UDP-glucuronate	0.78

^a^Fold change in total abundance comparing *S. pneumoniae* D39 in CDM supplemented with 30 μM Cd^2+^ relative to CDM alone.

^b^Did not meet statistical significance (*P* > 0.05).

### Disruption of glycolysis can be circumvented using the Pentose Phosphate Pathway

Manganese limitation in *S. pneumoniae* has previously been shown to influence glucose catabolism, diverting flux into the PPP^49,50^. This metabolic shift has been proposed to aid in mitigating the impact of decreased Mn^2+^-superoxide dismutase (SodA) expression and activity^49^ by regenerating NADPH consumed in maintaining the reduced glutathione pool of the cell. Here, our collective data are consistent with glucose flux into the PPP. We observed that, in addition to Cd^2+^-induced cellular depletion of Mn^2+^, *sodA* was significantly downregulated (−1.64-fold, *P* < 0.05) and the expressed protein was potentially mismetallated by Cd^2+^ (Table 2). In contrast to glycolysis, no genes in the PPP were downregulated by Cd^2+^ stress, and only transketolase (Tkt) was identified in the Cd^2+^-enriched peaks by metalloproteomics. Disruption of Tkt activity is predicted to be compensated for by the metal-independent alternative transketolase, TktCN (Fig. 4). Metabolomic analyses indicated glucose flux into the PPP with a significant increase in 6-phosphogluconate (6PG), a key PPP intermediate generated by glucose-6-phosphate 1-dehydrogenase (Zwf) during NADP^+^ reduction (*P* < 0.05, Table 4). Consistent with activation of this pathway, the metabolomics also showed that the glutathione pool was expanded in Cd^2+^-treated cells and predominantly present in the form of reduced glutathione. Collectively, these data are consistent with glucose flux into the PPP and provide a plausible pathway allowing the pneumococcus to circumvent the Cd^2+^-mediated blockages in the initial stages of glycolysis.

Glucose catabolism via the PPP can release F6P and GADP back to glycolysis, although given the putative impairment of PfkA and/or Fba, only GADP would be available for further consumption in Cd^2+^-treated cells (Fig. 4). However, the next key metabolic enzyme, glyceraldehyde 3-phosphate dehydrogenase (*gap*), has been shown to be readily inhibited by Cu^2+^ and Zn^2+^ ions^51,52^. Although Gap was not identified by metalloproteomics in the Cd^2+^-enriched fractions, it contains the same metal-binding residues as the Zn^2+^-susceptible Gap from *S. pyogenes* (92% identity)^51^, suggesting that it may be susceptible to Cd^2+^-mismetallation. However, *S. pneumoniae* also encodes an alternative, non-phosphorylating Gap variant, GapN, that can convert GADP to 3PG via the generation of NADPH, albeit at the expense of ATP production^53,54^. This alternate enzyme provides a pathway to circumvent inhibition of Gap and regenerate NADPH for maintenance of the expanded cellular glutathione pool. Consistent with this model, we observed that *gapN* was significantly upregulated (*P* < 0.05. Table 3) in Cd^2+^-treated *S. pneumoniae* compared to untreated. In addition, targeted metabolite analyses of Cd^2+^-treated cells revealed that cellular NADPH levels were approximately two-fold higher compared to untreated (*P* = 0.056), while ATP levels were significantly depleted (60%; *P* < 0.0001) in these cells (Supplementary Fig. 5). Thus, this adaptive metabolic response enables *S. pneumoniae* to utilize glucose during metal ion intoxication, but at the expense of ATP generation.

Collectively, these data provide insights into the way in which Cd^2+^ accumulation disrupts glucose metabolism leading to a reduction in the bacterial growth rate. Our findings reveal a multifactorial process involving depletion of cellular Mn^2+^, altered transcription of glycolytic pathway genes and possible Cd^2+^-mismetallation of glycolytic enzymes. However, this work also highlights how the pneumococcus can overcome these impediments to achieve growth in the presence of severe metal ion dyshomeostasis.

### Galactose does not protect *S. pneumoniae* from Cd^2+^-induced perturbation of energy production

Despite the primacy of glucose in *S. pneumoniae* carbon metabolism, the pneumococcus retains the capacity to utilize at least 32 other known sugars for energy production^55^. We observed that genes associated with galactose metabolism in the Leloir pathway were upregulated >2-fold (Table 3) in Cd^2+^-treated cells, by comparison with untreated. This was of particular interest as galactose utilization in *S. pyogenes* conferred resistance against Zn^2+^-intoxication^51^. In *S. pyogenes*, Zn^2+^-inhibited glycolysis at F6P and Gap, but this could be overcome by tagatose-6-phosphate pathway-mediated galactose catabolism. Accordingly, we investigated the capacity for galactose to protect *S. pneumoniae* from Cd^2+^-intoxication. This was addressed using a carbon-source free cation-defined media (CDM) supplemented with either 0.5% glucose (CDM-Glu) or 0.5% galactose (CDM-Gal). We observed that *S. pneumoniae* grown in CDM-Gal had reduced growth by comparison with CDM-Glu (Supplementary Fig. 1, Supplementary Table 1). Upon treatment with Cd^2+^, we observed that the maximal growth rates were highly similar for both carbon sources (0.54 for CDM-Glu vs. 0.46 for CDM-Gal h^−1^). This indicated that galactose was not protective against Cd^2+^. Unexpectedly, it suggested the Cd^2+^-intoxicated pneumococci metabolize both carbon sources at similar rates.

Carbon metabolism in *S. pneumoniae* can occur either via homolactic or mixed acid fermentation pathways. Catabolism of glucose and glycosaminoglycan disaccharides proceeds via homolactic fermentation, producing predominately lactate from pyruvate, via the action of lactate dehydrogenase (Ldh)^56,57^. In contrast, galactose is metabolized by mixed acid fermentation resulting in the production of acetate, formate, and ethanol^58^. The fermentation profile associated with galactose has been attributed to the slower metabolic flux of this sugar permitting carbon to be diverted to acetate production via the action of pyruvate formate-lyase which generates acetyl-CoA^59^. Metabolomic analyses of the terminal metabolic products of glucose in Cd^2+^-treated *S. pneumoniae* showed a significant reduction in lactate production, by comparison with untreated cells (Fig. 4 and Table 4). This occurred concomitantly with significant increases in the mixed acid fermentation end-products acetate (~15%, *P* = 0.0002) and ethanol (~250%, *P* = 0.0003) (Supplementary Fig. 5). Taken together, these data show that Cd^2+^ accumulation shifts the metabolic profile of glucose catabolism in *S. pneumoniae* from homolactic fermentation to mixed acid fermentation. This is most likely due to disruption of glycolysis and the impaired flux through FBP, which allosterically regulates Ldh activity. However, this catabolic pathway may play a crucial role for Cd^2+^-treated cells as it enables the generation of one ATP molecule per pyruvate molecule, through the concerted action of phosphotransacetylase (Pta) and acetate kinase (AckA) (Fig. 4).

**Fig. 5:**
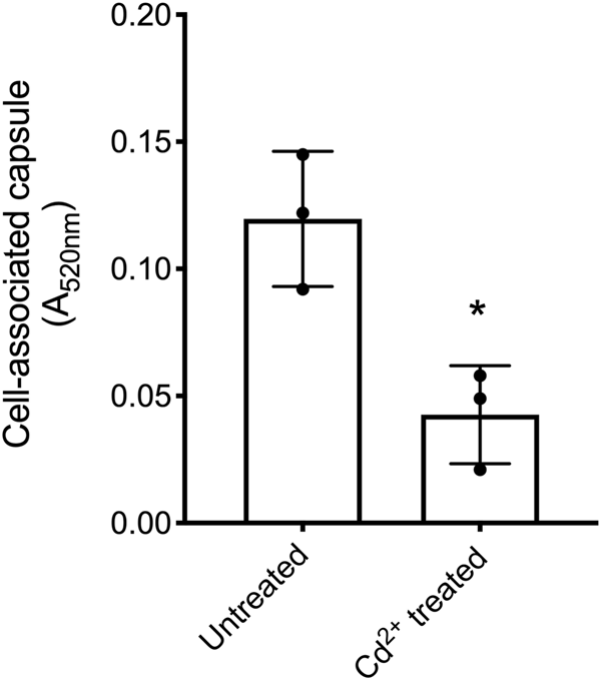
Capsule production during Cd^2+^-stress. Determination of capsule production via detection of uronic acid from *S. pneumoniae* D39 untreated and treated with 30 µM Cd^2+^. Data presented are the mean ± SD of three independent biological replicates (*n* = 3). The statistical significance of the differences in the mean data were determined by two-tailed unpaired *t*-tests (*P = 0.0153).

Although galactose catabolism by the Leloir pathway was not protective against Cd^2+^-induced perturbation of energy production, it may have other effects on *S. pneumoniae*. Capsule biosynthesis requires precursors, such as UDP-glucose and UDP-glucuronate, which may be affected by the upregulation of the Leloir pathway genes. Metabolomics revealed that the Leloir pathway intermediates, UDP-galactose, and galactose-1-phosphate showed significantly increased accumulation (*P* < 0.05) during growth in Cd^2+^-supplemented CDM (Table 4). This suggested that in Cd^2+^-treated cells, the upregulated Leloir pathway genes might be siphoning essential precursors away from capsule biosynthesis. We assayed pneumococcal capsule production via uronic acid detection and observed significantly (*P* < 0.05) less cell-associated capsule in Cd^2+^-treated cells, by comparison to untreated (Fig. 5). Taken together, these data suggest that activation of the Leloir pathway in response to cellular Cd^2+^ accumulation is not protective and results in reduced capsule production.

### Fatty acid biosynthesis is impaired during Cd^2+^ stress

Essential FAs required for the *S. pneumoniae* membrane are synthesized by the type II FA synthase (FASII) system^60^, which is encoded by the *fab* gene cluster (*fabMTHKDGFZ*). *De novo* FA biosynthesis also relies upon the acetyl-CoA carboxylase genes (*accBCDA*) and the acyl carrier protein (*acpP*), which is responsible for the transport of FAs. Transcriptional control of the FA biosynthetic gene cluster is primarily regulated by FA sensing of the negative regulator FabT, with FA biosynthesis genes upregulated in a Δ*fabT* strain^61^. We observed that *S. pneumoniae* lipid metabolism and FA biosynthesis were strongly affected by cellular Cd^2+^ accumulation with the FA biosynthesis genes downregulated between 3.3 and 6.7-fold, when compared to untreated cells (Table 3). Notably, expression of FabT was also reduced by 3.5-fold, suggesting that transcription of the *fab* cluster may be repressed via an alternative mechanism.

The first committed step in FA biosynthesis is the conversion of acetyl-CoA to malonyl-CoA. Metabolomic and transcriptomic analyses revealed a significant four-fold decrease in the abundance of acetyl CoA (*P* = 0.01) in Cd^2+^-treated cells, by comparison with untreated cells. This occurred concomitantly with a 19-fold increase in *adhC* transcription and increased ethanol production (Fig. 4 and Tables 3, 4). Conversion of acetyl CoA to malonyl CoA occurs via acetyl CoA carboxylase (AccBCDA), a heteromeric enzyme requiring biotin and Zn^2+^ cofactors^62^. We observed that Cd^2+^ exposure resulted in a two-fold decrease (*P* = 0.0025) in biotin attributable to the 3.6-fold downregulation of the biotin importer (*bioY*) (Tables 3, 4). Further, AccD, the Zn^2+^-dependent subunit of AccBCDA, was detected by metalloproteomics within a Cd^2+^-enriched peak (Table 2) suggesting mismetallation. Taken together, these data show that Cd^2+^-accumulation disrupts the FASII system at a transcriptional and functional level. This manifests through perturbation of the initial step in the FA biosynthesis pathway and a generalized downregulation of the FASII genes. Accordingly, we sought to assess the impact of this disruption on membrane composition.

### Membrane composition and fluidity is altered by Cd^2+^ stress

**Fig. 6:**
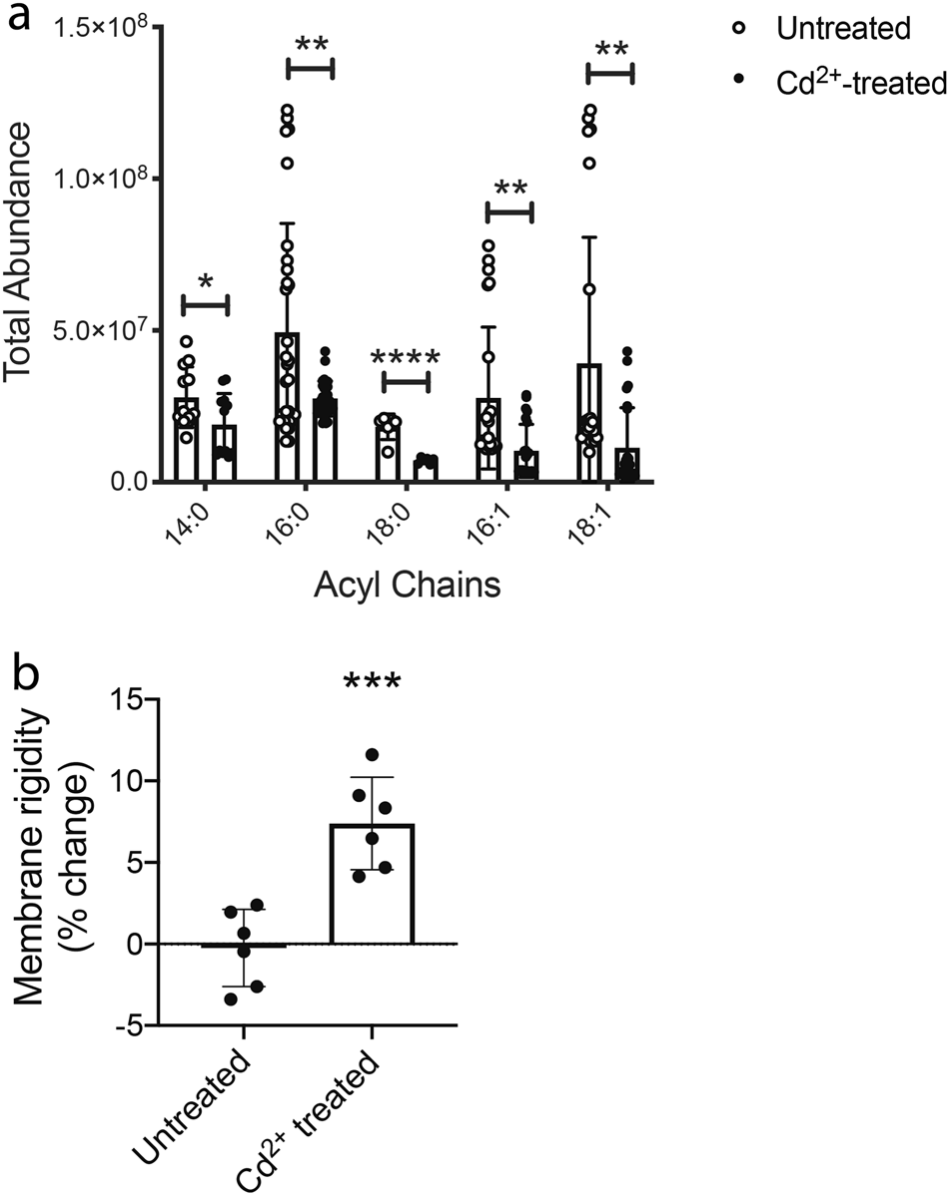
Cadmium induced changes to the *S. pneumoniae* membrane. Biophysical changes to the *S. pneumoniae* membrane in response to 30 µM Cd^2+^ stress. **a** Total abundance of saturated (14:0, 16:0, 18:0) and unsaturated (16:1, 18:1) acyl chains as determined by LC/MS metabolomic analyses. Acyl chain abundance in untreated *S. pneumoniae* is shown in white circles and acyl chain abundance from 30 µM Cd^2+^-treated *S. pneumoniae* is shown in black circles. Data presented are the mean ± SD of six independent biological replicates (*n* = 6). The statistical significance of the differences in the mean data were determined by two-tailed unpaired *t*-tests (*P = 0.0383, **P = 0.0016, 0.0013, 0.0029, and ****P < 0.0001). **b** Membrane rigidity as determined by diphenylhexatriene fluorescence normalized to untreated signal and expressed as % change. Data presented are the mean ± SD of six independent biological replicates (*n* = 6). The statistical significance of the differences in the mean data were determined by two-tailed unpaired *t*-tests (***P = 0.0005).

In addition to de novo FA biosynthesis by the FASII system, the pneumococcus can incorporate exogenous FAs into its membrane via FA phosphorylation using the FakAB1B2B3 and PlsXY systems^61,63,64^. This facilitates phospholipid acquisition without use of the energy intensive FASII system. We observed that expression of the *plsXY* and *fakAB1B2* systems were not affected by Cd^2+^ exposure. In contrast, *fakB3* (SPD_0646), which is responsible for incorporation of polyunsaturated FAs, was downregulated ~4-fold relative to untreated cells. This suggests acquisition of exogenous unsaturated FAs may be impaired during exposure to Cd^2+^ ^63^. Thus, we next examined acyl chain composition of the *S. pneumoniae* membrane via LC-MS. In Cd^2+^-treated cells, the abundance of saturated acyl chains (14:0, 16:0 and 18:0) was reduced 1.4–2.5-fold and monounsaturated acyl chains (16:1 and 18:1) were extensively depleted (2.7–3.4-fold) (Fig. 6a). The physiological impact of the increased abundance of saturated acyl chains, relative to unsaturated acyl chains, on the pneumococcal membrane was then assessed. Using the fluorescent probe diphenylhexatriene, we observed that Cd^2+^-treated cells had significantly increased membrane rigidity relative to untreated cells (*P* = 0.0005) (Fig. 6b). This indicated that the greater ratio of saturated to unsaturated acyl chains resulted in tighter packing of saturated phospholipids in the cell membrane during metal ion dyshomeostasis.

## Discussion

Transition metal ion homeostasis is crucial for the viability of all organisms. In the human pathogen *S. pneumoniae*, we have probed the molecular and cellular impacts of metal ion dysregulation by using the non-physiological metal ion Cd^2+^. This exploits the extensive chemical similarity of Cd^2+^ with the biologically essential metal ion Zn^2+^ ^8,12^. However, the pneumococcus lacks Cd^2+^-specific regulatory and efflux pathways most likely due to limited environmental exposure and an absence of selective pressures to evolve resistance. As a consequence, Cd^2+^ is readily accumulated by *S. pneumoniae* resulting in dysregulation of Mn^2+^ and Zn^2+^ homeostasis mechanisms^18^. Thus, Cd^2+^ is an ideal probe to study the broader cellular impacts that arise from disruptions in metal ion homeostasis. Although the core pathways and processes impacted by Cd^2+^ stress in *S. pneumoniae* are conserved in many bacteria, we predict that as organism complexity increases as a function of genome size, this will provide both additional mechanisms of resistance to Cd^2+^ and targets of susceptibility.

The FASII biosynthetic pathway and the exogenous FA acquisition systems are essential for membrane biogenesis in *S. pneumoniae*^65^. Our investigations showed that exposure to Cd^2+^ was associated with a downregulation of the genes in the FASII pathway, albeit via an unknown mechanism. Studies in other bacteria, notably *E. coli*^66^ and *B. subtilis*^67^, have previously shown that *acc* gene transcription, and therefore the first committed step in the FASII pathway, is directly related to the growth rate of the organism. Although this has not been established to occur in *S. pneumoniae*, the Cd^2+^-induced reduction in the growth rate may impact the expression of the FASII pathway. Alternatively, downregulation of the FASII pathway may be due to an additional regulatory mechanism, such as YycFG, which has been found to modulate the expression of the FASII system in *S. pneumoniae*^68,69^, and was not transcriptionally impacted by Cd^2+^ exposure. Although further studies will be required to elucidate the precise mechanisms for how these biosynthetic pathways are impacted, our findings highlight how Cd^2+^-induced changes in central carbon metabolism directly influence other essential cellular biosynthetic pathways.

The effect of Cd^2+^ treatment on phospholipid production resulted in significant changes to membrane composition. Notably, an increase in membrane rigidity due to the greater ratio of saturated:unsaturated membrane acyl chains. Unsaturated FAs contain double carbon bonds that are highly susceptible to reactive oxygen species such as H_2_O_2_ and OH⦁^70^. Prior studies have shown that exposure of *S. pneumoniae* to H_2_O_2_ resulted in a reduction in the abundance of double bonds within the membrane phospholipids^71^. This has been attributed to FabF, which has a thiol-reactive cysteine that serves as a sensor of cellular oxidative stress^72^. Accordingly, in Cd^2+^-treated *S. pneumoniae* the depletion of cellular Mn^2+^ and concomitant loss of Mn-SOD may serve as a trigger for shifting the ratio of saturated:unsaturated FAs in the membrane in order to minimize susceptibility to lipid peroxidation^73^. Thus, the physiological consequence of this change is decreased membrane fluidity and increased resistance to intracellular oxidative stress.

Central carbon metabolism was prominently impacted by Cd^2+^ accumulation with multiple disruptions in glycolysis, activation of the PPP, and a switch to mixed acid fermentation of pyruvate. Glucose is primarily catabolized by glycolysis in *S. pneumoniae*. However, glucose flux can be diverted into the PPP by depletion of NADPH, as shown in *Corynbacterium glutamicum*^74^. Cellular depletion of Mn^2+^, which occurs in *S. pneumoniae* during Cd^2+^ stress^18^, also activates this metabolic switch to enable regeneration of NADPH required to maintain the cellular pool of reduced GSH^49^. This is crucial for Mn-SOD independent resistance against oxidative stress and, during exposure to Cd^2+^, for expansion of the reduced GSH pool to buffer intracellular Cd^2+^ ions. Thus, we infer that activation of the PPP is an adaptive metabolic change that enhances resistance to metal stress, regulated via NADPH abundance. Further, we speculate that the PPP is finely tuned to be a metal-intoxication resistant pathway, comprised of metal ion independent catabolic enzymes such as Zwf, Gnd, RpiA, TalC and TktCN^75–77^. In this way, the PPP enables glucose catabolism in the presence of metal ion intoxication, albeit with less efficiency than the metalloenzyme-dominated pathway of glycolysis, with metabolites entering the latter half of glycolysis and continuing their catabolic progression via metal-independent alternative enzymes such as GapN^53^. Hence, this work highlights the evolutionary cost of employing metal-dependent proteins. Although metal ions can be used to enhance protein activity and thereby enable more efficient enzymatic functionality, as exemplified in glycolysis, this dependency renders metalloproteins susceptible to inactivation upon interaction with non-cognate metals, such as Cd^2+^. Nevertheless, we cannot exclude the possibility that activation of the PPP arises from impaired flux through glycolysis due to downregulation of glycolytic genes, possibly as a result of the Cd^2+^-induced growth delay, and/or impaired function of glycolytic metalloenzymes. In the absence of Cd^2+^ the glycolytic metabolite FBP regulates the direction of carbon flux via activation of the enzymes PfkA and Pyk and inhibition of Gnd, which prevents flux into the PPP^74,78,79^. FBP also allosterically activates Ldh and thereby regulates the fermentation pathway of pyruvate^80^. Treatment of *S. pneumoniae* with Cd^2+^ reduced the abundance of FBP relative to other glycolytic intermediates, attributable to decreased expression of *pfkA* and potential Cd^2+^-mismetallation of the enzyme. As a consequence, this impairment in glycolysis may also trigger activation of the PPP. Irrespective of the mechanistic basis, glucose appears to be catabolized by the PPP during Cd^2+^-induced metal ion dyshomeostasis enabling regeneration of NADPH, but at the expense of ATP production. The overall rate of glucose flux through the PPP appears to be lower than that observed with glycolysis as suggested by the increased abundance of metabolic intermediates from the latter half of glycolysis. Despite the primacy of CcpA in regulating optimal carbon catabolism in *S. pneumoniae*^48,81^, the observed transcriptional changes in key metabolic genes appear inconsistent with CcpA-dependent regulation of glucose catabolism^48^. However, it is important to note that studies of CcpA in *S. pneumoniae* have used a variety of distinct nutritional parameters^48,81^. The resultant differences in growth rates, gene expression and analytical approaches complicate direct comparisons between studies. Nevertheless, our findings suggest that the observed shunt toward the PPP is not coordinated by a general carbon catabolite repression mechanism, but is instead an adaptive response to the cellular impacts of Cd^2+^ exposure. Collectively, these observations highlight the capacity of *S. pneumoniae* to undergo an adaptative metabolic change in response to environmental stress.

The Cd^2+^-mediated impact on carbon flux also provides a mechanistic explanation for how intracellular metal abundance can directly influence biosynthetic pathways, such as capsule production. In *Lactococcus acidophilus* it has been shown that Fe^2+^ and Cu^2+^ supplementation increases the production of the primary capsule component hyaluronic acid (HA) via interaction with PPP enzymes and pathway intermediates^82^. In contrast, Zn^2+^ intoxication of *S. pyogenes* decreased HA capsule production due to inhibition of phosphoglucomutase (Pgm)^51^. Here, we observed that Cd^2+^accumulation also resulted in decreased capsule levels, although this appeared to be independent of *pgm* expression and function. This indicated an alternative mechanism of capsule biosynthesis disruption in Cd^2+^-treated pneumococci. A possible explanation is that inappropriate activation of the Leloir pathway during Cd^2+^ exposure results in siphoning of αGlu1P, thereby preventing its flux into capsular polysaccharide production and leading to the reduced capsule.

Collectively, this study has revealed the extensive breadth of cellular and metabolic adaptations required for the pneumococcus to maintain viability during metal ion dyshomeostasis. The accumulation of Cd^2+^ in the cytoplasm exerts substantial deleterious impacts upon the cell by disrupting energy production, likely through a combination of cellular Mn^2+^ and Zn^2+^ depletion and possible competition between Cd^2+^ and native metal cofactors for crucial metalloproteins. Importantly, this work has shown how adaptive changes in central carbon metabolism may be used to mitigate the loss of metal ion homeostasis and provide continued energy production during metal ion intoxication.

## Methods

### Growth of *S. pneumoniae* D39

*S. pneumoniae* was routinely grown in cation-defined media (CDM), which corresponded to C + Y media without supplementation of transition metals^83^. The base transition metal concentration of the media was determined by ICP-MS on an Agilent 7500cx (Adelaide Microscopy, University of Adelaide) to be: 0.23 µM manganese, 5.6 µM iron, 0.15 µM cobalt, 0.16 µM nickel, 2.8 µM copper, and 12.1 µM zinc. CDM was routinely prepared with 0.2% [w/v] glucose for microbiological analyses^83^. Carbon-source comparison growth assays were conducted in 0.5% [w/v] glucose or 0.5% [w/v] galactose where specified. All growth experiments were conducted in CDM supplemented with 1 μM MnSO_4_. Thirty micromolar CdCl_2_ was added where specified. All chemicals used in this study were purchased from Sigma Aldrich unless otherwise specified. Cultures of *S. pneumoniae* D39 were routinely prepared from overnight growth on blood agar, resuspended, and inoculated into CDM to an absorbance at 600 nm (*A*_600_) of 0.05. The culture was incubated at 37 °C + 5% CO_2_ and grown to *A*_600_ = 0.3. Growth kinetic assays were conducted in 96-well plate format using a FLUOstar Omega spectrophotometer (BMG Labtech). *S. pneumoniae* were inoculated in a final volume of 200 μL CDM (± supplementation where indicated) to a starting *A*_600_ = 0.01 in a clear 96-well plate (Greiner). Plate was incubated at 37 °C + 5% CO_2_ for >16 h with readings taken every 30 min. Growth assay data were analyzed using GraphPad Prism.

### RNA sequencing and qRT-PCR sample preparation

*S. pneumoniae* was grown as detailed above. At mid-log phase (*A*_600_ = 0.3), 500 μL of culture was mixed with 1 mL of RNA Protect (Qiagen) and cells were harvested via centrifugation before storage at −80 °C. Bacterial pellets were RNA extracted and purified using RNeasy Protect Bacterial Mini kit (Qiagen) after enzymatic lysis using lysozyme and mutanolysin, all according to the manufacturer’s instructions. DNase treatment was performed on-column during RNA extraction using RNase-free DNase (Qiagen).

### qRT-PCR

Quantitative reverse transcription PCR was conducted using Superscript III (Invitrogen) on a QuantStudio 7 Real-time PCR system (Applied Biosystems). The transcription levels of genes analyzed were normalized to those obtained for 16s rRNA. Primer sequences are listed in Supplementary Table S2.

### RNA sequencing

RNA was extracted and prepared as above from biological quadruplicates of *S. pneumoniae* D39. RNA was pooled and analyzed on a Bioanalyzer 2100 (Agilent) to confirm a RIN value > 8 according to the manufacturer’s instructions. RNA was then submitted to the Australian Genome Research Facility (AGRF) for sequencing. In brief, the Epicentre Bacterial Ribozero Kit (Illumina) was used to deplete ribosomal RNA content before the generation of barcoded libraries using Ultra Directional RNA kit (New England Biolabs). Prepared libraries were then sequenced using an Illumina HiSeq2500 with Version 3 SBS reagents and 2 × 100 bp single-end chemistry. Reads were aligned to the *S. pneumoniae* D39 genome (GenBank accession number NC_008533) using BOWTIE2 version 2.2.6^84^. Counts for each gene were obtained using SAMtools version 1.2^85^ and BEDtools version 2.24.0^86^, and differential gene expression was determined using R (DESeq Library) version 3.2.2^87^. Transcriptomic data have been deposited in the NCBI Gene Expression Omnibus databank under submission identifier GSE141681.

### Bioinformatic COG analysis

Clusters of Orthologous Groups (COG) analysis was performed using the *S. pneumoniae* D39 reference sequence (NC_008533.gbk) from the National Centre for Biotechnology Information (NCBI). COG classifications of proteins (as designated by NCBI) are applied based on computational analysis of sequenced genomes to predict protein functionality and/or biochemical behavior based on homology to currently characterized proteins^88^. All available COG classifications for the *S. pneumoniae* D39 genome were aligned to RNA sequencing data and clustered according to the functional category. Calculations were then performed to determine the percentage of genes in each cluster that showed differential expression of greater than two-fold.

### Metabolomic sample preparation

*S. pneumoniae* cultures for metabolomic analyses were grown as detailed above in biological sextuplicate. At *A*_600_ = 0.3, cultures were harvested by centrifugation at 7000 × *g* for 7 mins at 4 °C. Once pelleted, cells were flash-frozen using liquid nitrogen to preserve the metabolite profile and stored at −80 °C. Cultures were processed and analyzed via LC/MS by Metabolon, North Carolina, USA.

### Targeted metabolite analyses

For metabolites that were not detected using metabolomics, targeted analyses using commercially available kits were undertaken for the detection of ethanol (Megazyme), acetate (Biovision), ATP (BacTiter-Glo; Promega) and NAD(P)H/NAD(P) (Abcam). All assays were carried out according to the manufacturer’s instructions. In brief, *S. pneumoniae* were grown in 7 mL cultures of CDM ± 30 µM Cd^2+^ as previously described in a minimum of biological triplicate. Cells were harvested via centrifugation at 7000 × *g* for 7 min at 4 °C and resuspended in assay-appropriate buffer. All statistical analyses were conducted using a two-tailed, unpaired *t*-test (GraphPad Prism).

### Metalloproteomic sample preparation

*S. pneumoniae* cultures for metalloproteomic analyses were prepared as detailed above. At *A*_600_ = 0.3, 45 mL cultures were harvested by centrifugation at 4 °C for 7 min at 7000 × *g*. Cell pellets were then washed thrice with 20 mL PSB, 5 mM EDTA, followed by three washes with 20 mL PBS at 4 °C for 7 min at 7000 × *g*. The harvested cell pellets were then stored at −80 °C for LC-ICP-MS analysis.

### LC-ICP-MS

Metalloproteomic culture cell pellets were resuspended in 200 μL 20 mM Tris-HCl pH 8.0 and sonicated using a Bioruptor system (Diagenode) for 25 cycles (30 s on, 30 s off). Sonicated cultures were then centrifuged at 4 °C for 15 min at 18,000 × *g* to remove insoluble material. The supernatant was harvested and 2 mg total protein was fractionated via AEXchromatography using a Bio IEX 3 μm column (Agilent) on an Infinity 1260 HPLC system (Agilent). AEX fractions were collected in 1-min intervals, corresponding to 400 μL fractions. AEX fractions were then independently separated via SEC using a Bio SEC-3 column (Agilent) on an Infinity 1260 HPLC system (Agilent), directly hyphenated to an ICP-MS 7500cx (Agilent) to determine metal content. Detection of metals (recorded in counts per second) were normalized to an internal standard (antinomy [Sb]) and metal concentration (in ppb) was interpolated from a calibration curve of known metal concentrations. Determination of protein-metal interactions (denoted by number of peaks with increased metal abundance) was conducted using baseline-corrected area under curve analysis (GraphPad Prism 8.0). Metalloproteomic maps were generated using MATLAB 2020a.

### Mass spectrometry

MS samples were prepared essentially according to^89^. In brief, selected AEX fractions were chosen for MS analysis based on the presence of Cd^2+^ and Zn^2+^ peaks in the metalloproteomic maps. The protein concentrations of the chosen AEX fractions were determined by *A*_280_. Approximately 0.5 μg of protein was added to an equal volume of HPLC grade acetonitrile (ACN), and trypsin digested according to protocol^89^. Proteins were harvested through evaporation of the liquid phase in a vacuum concentrator centrifuge for ~2 h. Samples were individually resuspended in 250 μL of 0.1% v/v trifluoroacetic acid (TFA) in ddH_2_O, and C18 treated using the default ‘Peptide Clean-up’ method on the automated BRAVO liquid handling robot (Agilent). Eluted peptides were harvested through evaporation of the liquid phase and resuspended in 10 μL 2% v/v ACN, 0.1% v/v TFA in ddH_2_0. Final protein concentration was determined by *A*_280_, with all samples having a final concentration of ~1.5 mg mL^−1^.

Samples were then run on the Q Exactive^TM^ Plus Hybrid Quadrupole-Orbitrap^TM^ Mass Spectrometer (ThermoFisher Scientific) at the Bio21 Institute, Melbourne, Australia.

### MS data analysis

MS data analysis was conducted using Matrix Scientific MASCOT Server. Data files (.mgf) were uploaded into MASCOT MS/MS ion search and analyzed using SwissProt database for matches to *Streptococcus pneumoniae* taxonomy. Parameters were as follows: Enzymatic cleavage by trypsin, allowing one missed cleavage site. Fixed Carbamidomethyl (C) modification and variable Oxidation (M) modification. Peptide tolerance of 1.2 Da, MS/MS tolerance of 0.6 Da with monoisotopic mass values. Peptide charges of +1, +2, +3 were allowed with an unrestricted protein mass. The significance threshold was set at *P* < 0.05. Ion scores of ≥24 were indicative of protein identity or extensive homology. Only proteins with >2 peptide hits with ion scores ≥ 24 were considered true identifications in accordance with^90^.

### Uronic acid capsule assay

*S. pneumoniae* was grown as previously described to a final *A*_600_ = 0.2, harvested via centrifugation and resuspended in 500 µL 150 mM Tris-HCl pH 7.0, 1 mM MgSO_4_. Subsequent sample processing and quantification of capsular uronic acid was conducted as described in refs. ^91,92^. In brief, resuspended cultures were incubated at 37 °C for 30 min with 0.01% [v/v] deoxycholate to lyse the cells, followed by overnight incubation at 37 °C with 100 U mutanolysin, 50 µg DNase and 25 µg RNase. Samples were then subsequently incubated with 100 µg proteinase K for 4 h at 56 °C. One hundred microlitres of each sample were then added to 600 µL 12.5 mM sodium tetraborate in 98% [v/v] H_2_SO_4_. Samples were vortexed and heated at 95 °C for 5 min and cooled on ice before the addition of 3-phenylphenol solution (10 µL). Samples were then immediately added to a clear 96-well plate and absorbance was read at 520 nm using a PHERAstar spectrophotometer (BMG Labtech). The amount of cell-associated capsule was determined from 3 independent experiments and the statistical difference was assessed by a two-tailed, unpaired *t*-test (GraphPad Prism).

### Membrane rigidity assay

Membrane rigidity was determined as described in^93^. In brief, bacterial cultures were grown as previously described to *A*_600_ = 0.3 and cells were washed in PBS. Bacteria were incubated with 1,6-diphenyl-1,3,5-hexatriene dissolved in tetrahydrofuran. After incubation at 37 °C for 30 min, cells were washed and fluorescence polarization (excitation 350/emission 450 nm) was determined on a PHERAstar spectrophotometer (BMG Labtech). The relative change in membrane rigidity was determined from six independent experiments and the statistical difference was assessed by a two-tailed, unpaired *t*-test (GraphPad Prism).

### Statistics and reproducibility

Sample sizes were derived based on the accepted conventions for life sciences research, i.e., for independent biological replicates, *n* = 3 is sufficient for performing statistical comparisons between experimental groups. Experiments contributing to a single dataset were performed across multiple days/weeks to ensure that analyses were being conducted across independent bacterial cultures and batches of reagents. For the -omics analyses, sample sizes were guided by acceptable industry standards. RNA sequencing used four pooled biological replicates (Australian Genome Research Facility), metabolomic analyses used six biological replicates (Metabolon), metalloproteomic maps and MS data were generated from single biological replicates to allow direct comparison between metal ion determination and protein identity. Data reproducibility was ensured through temporal separation of replicate experiments.

All statistical analyses were conducted using GraphPad Prism. Statistical tests were selected based on the nature of the data. For direct comparison of untreated and Cd^2+^-treated groups, two-tailed, unpaired *t*-tests were routinely applied.

### Reporting summary

Further information on research design is available in the Nature Research Reporting Summary linked to this article.

## Supplementary information

Supplementary Information

Description of Additional Supplementary Files

Supplementary Data 1

Supplementary Data 2

Reporting Summary

## Data Availability

RNA sequencing data have been deposited in NCBI Gene Expression Omnibus databank under submission identifier GSE141681. The mass spectrometry proteomics data have been deposited to the ProteomeXchange Consortium via the PRIDE^94^ partner repository with the dataset identifier PXD021643 and 10.6019/PXD021643. Raw data files for all other Figures are available from the corresponding authors upon reasonable request.
