# Supplementary material for: Cadmium stress dictates central carbon flux and alters membrane composition in Streptococcus pneumoniae
Source: Commun Biol. 2020 Nov 19;3:694. doi: 10.1038/s42003-020-01417-y (PMC7678824; doi:10.1038/s42003-020-01417-y)
Supplement: Supplementary file 1 — Supplementary Information [file 42003_2020_1417_MOESM1_ESM.pdf]

Cadmium stress dictates central carbon flux and alters membrane composition in *Streptococcus pneumoniae*

Stephanie L. Neville [1*], Bart A. Eijkelkamp [2], Amber Lothian [3], James C. Paton [4], Blaine R. Roberts [3,5], Jason W. Rosch [6] and Christopher A. McDevitt [1*]

1. Department of Microbiology and Immunology, The Peter Doherty Institute for Infection and Immunity, The University of Melbourne, Melbourne, Victoria, Australia.

2. College of Science and Engineering, Flinders University, Bedford Park, South Australia, Australia.

3. Melbourne Dementia Research Centre, The Florey Institute of Neuroscience and Mental Health, The University of Melbourne, Parkville, Victoria, Australia.

4. Research Centre for Infectious Diseases, Department of Molecular and Biomedical Science, University of Adelaide, Adelaide, South Australia, Australia.

5. Department of Biochemistry, Emory University School of Medicine, Atlanta, GA 30322 USA

6. Department of Infectious Diseases, St Jude Children's Research Hospital, Memphis, Tennessee, USA.

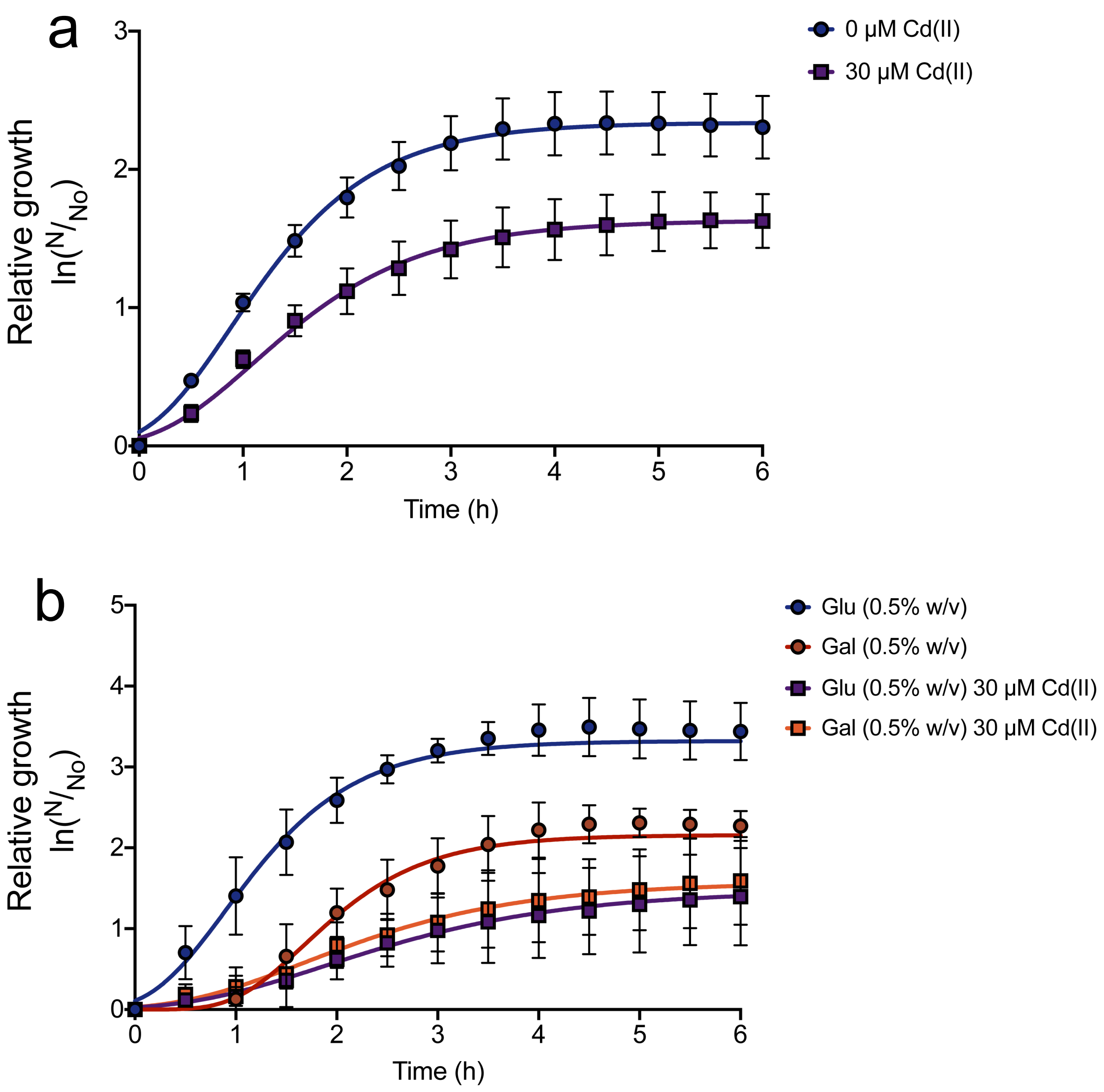

Supplementary Figure 1: Growth kinetics of *S. pneumoniae*.

(a) Growth kinetics of *S. pneumoniae* grown in standard CDM (0.2% [w/v] glucose). Untreated (0 μM Cd(II)) in blue circles and Cd^{2+}-treated (30 μM Cd(II)) in purple squares. (**b**) Growth kinetics comparing *S. pneumoniae* grown with either 0.5% [w/v] glucose (Glu) or 0.5% [w/v] galactose (Gal) as the sole carbon source in untreated conditions (blue and red circles, respectfully), or in the presence of 30 Cd^{2+} (purple squares- Glu, and orange squares- Gal). Data presented represents the mean ± SD of biological triplicate (n=3).

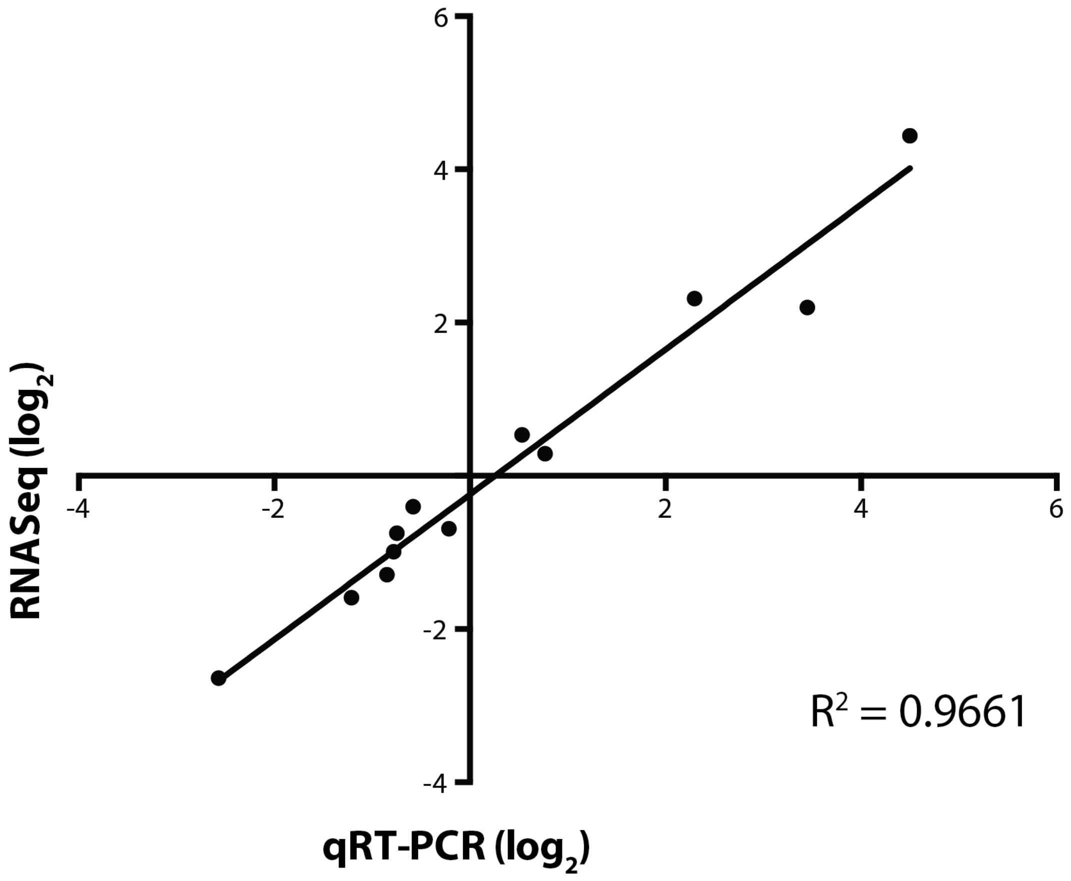

Supplementary Figure 2: Validation of RNA sequencing with qRT-PCR:

The differential expression of 12 genes were compared between RNA sequencing (x-axis) and qRT-PCR (y-axis) of mid-log phase *S. pneumoniae*. Gene expression changes are presented as log_2 fold-change. Genes included for comparison were: SPD_0150, SPD_0384, SPD_0685, SPD_0889, SPD_0890, SPD_0915, SPD_1384, SPD_1450, SPD_1463, SPD_1638, SPD_1652 and SPD_1927.

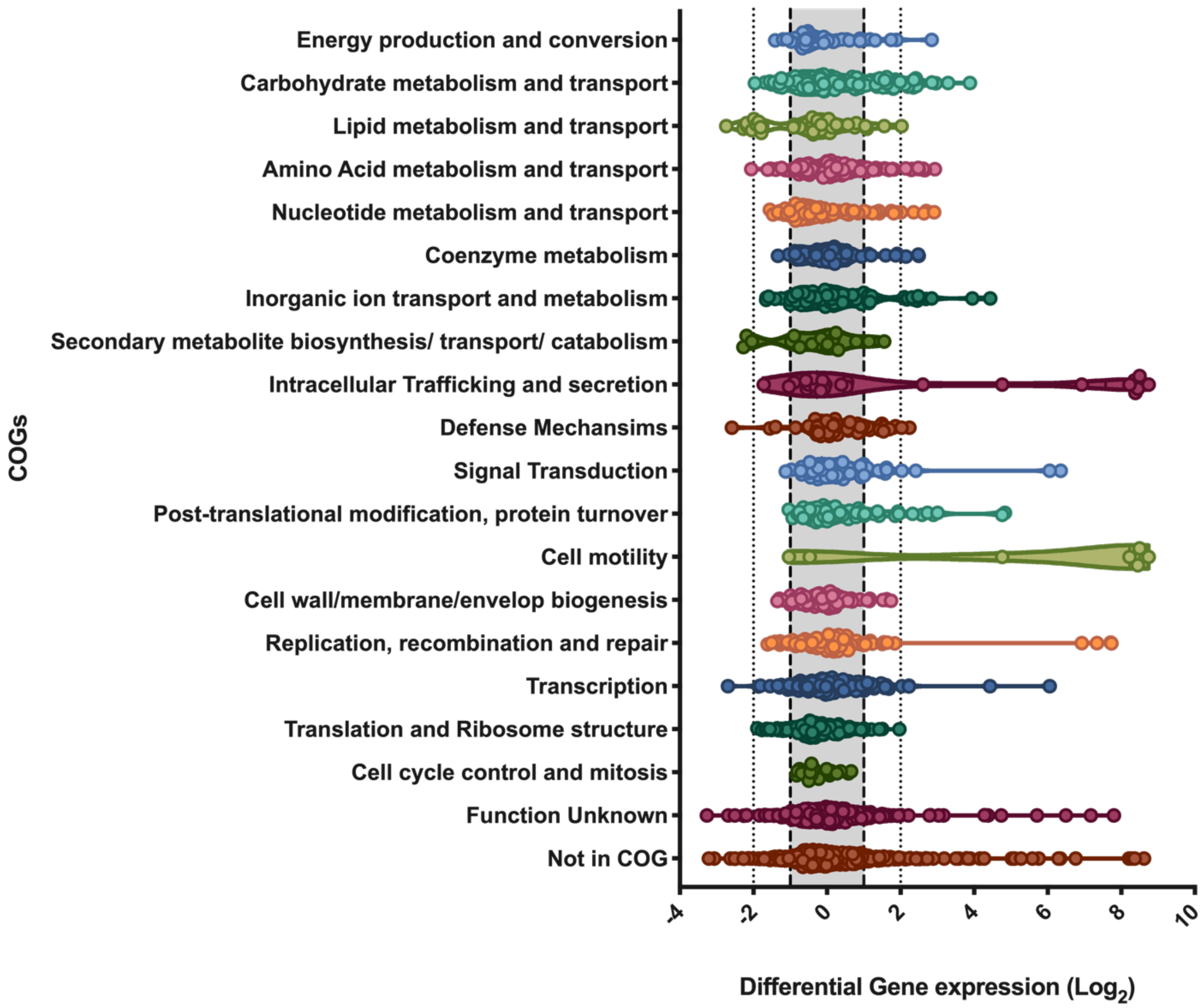

Supplementary Figure 3: Clusters of Othologous Genes analysis of transcriptome data

Log_2 fold-change of gene expression in *S. pneumoniae* treated with 30 µM Cd^{2+} compared to untreated. Each circle represents an individual gene with the shaded areas illustrating violin plots of the frequency distribution of the data.

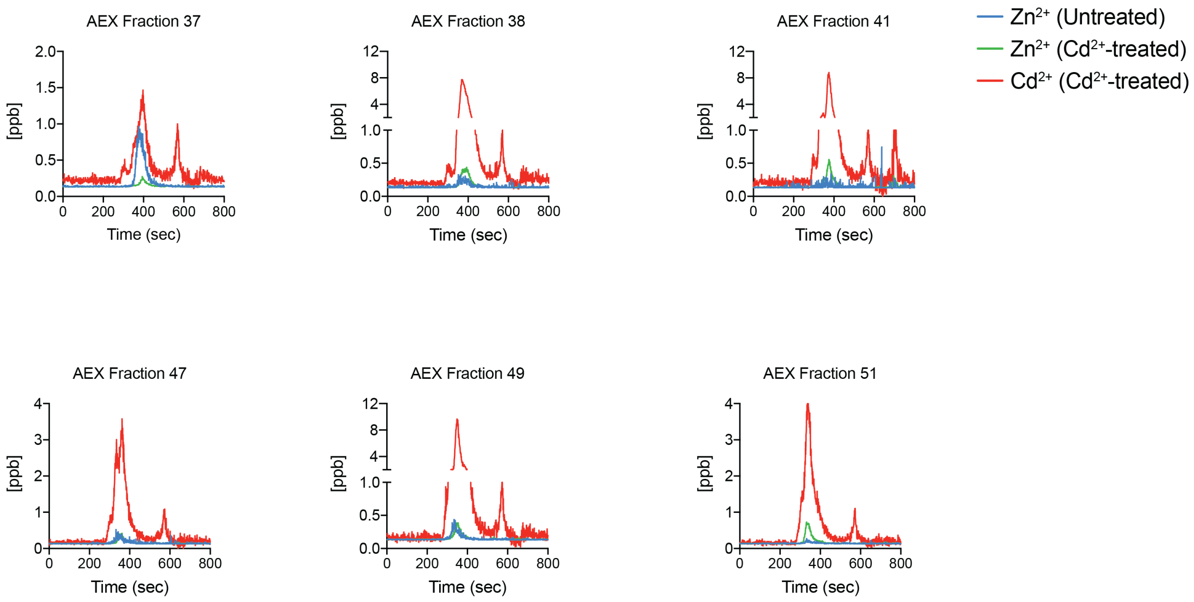

Supplementary Figure 4: Comparison of LC-ICP-MS protein-associated metal peaks

Representative anion exchange (AEX) fractions during size exclusion separation of the *S. pneumoniae* cytoplasmic proteome as detected by ICP-MS. Zinc trace from untreated *S. pneumoniae* (blue) has been overlayed with the Zn^{2+} trace from Cd^{2+}-treated pneumococci (green) and the Cd^{2+} trace from Cd^{2+}-treated pneumococci (red). The metal detected by the ICP-MS is presented on the y-axis as metal concentration (in parts per billion [ppb]). Proteins identified by mass spectrometry in the above fractions have been reported in Table 2 and Supplementary Data 1.

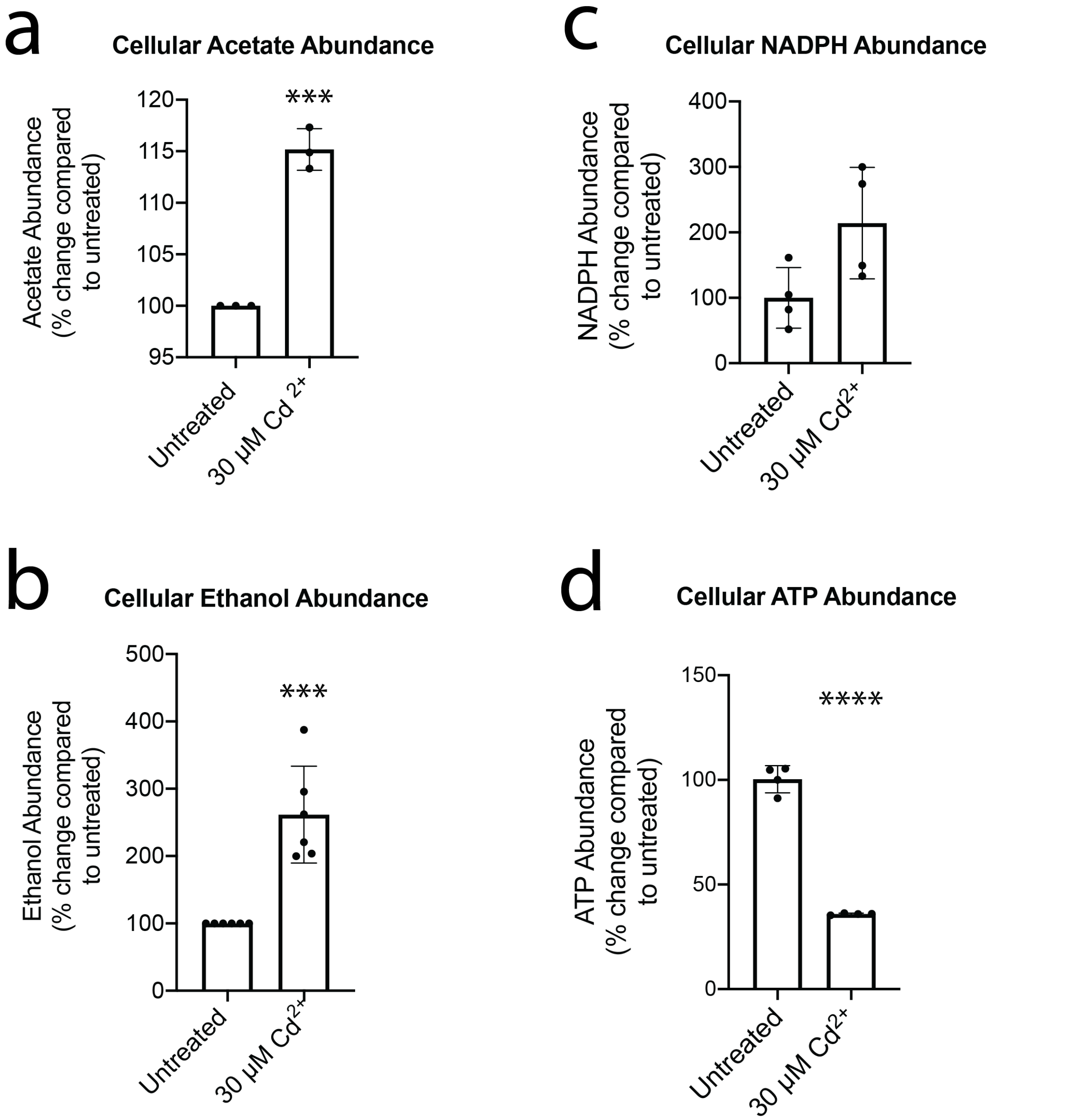

Supplementary Figure 5: Targeted metabolite analysis of Cd^{2+}-treated *S. pneumoniae*
Cellular abundance of acetate **(a)** ethanol **(b)** NADPH **(c)** and ATP **(d)** in response to growth in the presence of 30 µM Cd^{2+}compared to untreated cultures. Untreated samples have been normalised to 100% and the % change has been calculated for Cd^{2+}-treated samples. Data points represent independent biological replicates. Error bars show mean ± SD. The statistical significance of the differences in the data were determined by two-tailed unpaired *t*-tests (****P*=0.0002 [acetate], *** *P*=0.0003 [ethanol], not significant [NADPH], **** *P*<0.0001 [ATP]).

Supplementary Table 1. Parameter values for the modified Gompertz model for *S. pneumoniae*

**Strain and Growth Condition**	**μmax (h^{-1})**	**λ (h)**	**N_{max}**	**R^2**
D39 1 μM Mn^{2+}	1.109	0.114	2.337	0.9972
D39 1 μM Mn^{2+}, 30 μM Cd^{2+}	0.661	0.190	1.631	0.9992
D39 1 μM Mn^{2+}, 0.5% glucose	1.680	0.169	3.322	0.9739
D39 1 μM Mn^{2+}, 30 μM Cd^{2+}, 0.5% glucose	0.543	0.404	1.470	0.9878
D39 1 μM Mn^{2+}, 0.5% galactose	1.156	0.987	2.159	0.9521
D39 1 μM Mn^{2+}, 30 μM Cd^{2+}, 0.5% galactose	0.462	0.477	1.573	0.9605

Supplementary Table 2: Primers used in this study

Primer Name	Primer Sequence (5'-3')
SPD_0150_F	TTGCTGCCCTAGGACTTGTT
SPD_0150_R	CCAGTCAATTCGCCATTTTC
SPD_0384_F	GTTCGAGTCGTGGAATTGGT
SPD_0384_R	TCTCCTGAAATGGGAACCAC
SPD_0685_F	AGTTCCGATAGCAGGGTGTG
SPD_0685_R	GATGTAACGGGCGAGAAAGA
SPD_0889_F	GTATTAGACAAAATGCTGTGGAG
SPD_0889_R	CTGTATAGGAGTCGGTTGACTTTC
SPD_0890_F	GGAACAGTTGAGAACCAACCA
SPD_0890_R	TAGGGTCACTCCCCACATTC
SPD_0915_F	AGCGCTTGTTCTTCTAATTCTG
SPD_0915_R	ACATCATGATTTCCCCAAGC
SPD_1384_F	TCAAGTTAGCTGAGCGTGGA
SPD_1384_R	TAAGAGGGCCACATTTCCAA
SPD_1450_F	TATTCCTGCCAAGGGAGAAC
SPD_1450_R	CTGGTCACCGATGTGAAGTG
SPD_1463_F	AGCCTATGGTGTTCCAAGTG
SPD_1463_R	GTTTTCATTGGACGGTCATC
SPD_1638_F	TTGGTTCTAGCGCTGTTCTT
SPD_1638_R	AGGCTCCTAGCAGGCTAAAC
SPD_1652_F	ATCCTTAGCAAGTGCCTTCG
SPD_1652_R	AAAGGCTGCCATTTTTCCTT
SPD_1927_F	GGAGAGTTACGCATTCGTGT
SPD_1927_R	CCCAGACCCCAAGAAATTAT

Supplementary Table 3. Differential expression of genes associated inorganic ion homeostasis

Locus tag	Gene/Locus	Predicted function	Log_2-fold change[a]
**Increased relative expression**			
SPD_0223	*pitC*	Fe^{3+} ABC transporter, transmembrane protein	+1.04
SPD_0224	*pitB*	Fe^{3+} ABC transporter, transmembrane protein	+1.00
SPD_0225	*pitD*	Fe^{3+} ABC transporter, ATP-binding protein	+1.03
SPD_0226	*pitA*	Fe^{3+} ABC transporter, solute-binding protein	+1.10
SPD_1461	*psaB*	Mn^{2+} ABC transporter, ATP-binding protein	+2.10
SPD_1462	*psaC*	Mn^{2+} ABC transporter, transmembrane protein	+2.18
SPD_1463	*psaA*	Mn^{2+} ABC transporter, substrate binding protein	+2.20
SPD_1638	*czcD*	Cation diffusion facilitator protein	+4.44
SPD_1649	*piuB*	Fe^{3+}-dicitrate ABC transporter, transmembrane protein	+2.36
SPD_1650	*piuC*	Fe^{3+}-dicitrate ABC transporter, transmembrane protein	+2.45
SPD_1651	*piuD*	Fe^{3+}-dicitrate ABC transporter, ATP-binding protein	+2.52
SPD_1652	*piuA*	Fe^{3+}-dicitrate ABC transporter, solute-binding protein	+2.32
**Decreased relative expression**			
SPD_0888	*adcAII*	Zn^{2+} ABC transporter, solute-binding protein	-1.24
SPD_0889	*phtD*	Zn^{2+}-binding histidine triad protein D	-1.29
SPD_0890	*phtE*	Zn^{2+}-binding histidine triad protein E	-2.62
SPD_0789	*piaB*	Ferrichrome siderophore ABC transporter, transmembrane protein	-1.00
SPD_1037	*phtB*	Zn^{2+}-binding histidine triad protein B	-1.30
SPD_1588	*SPD_1588*	Putative Hemin transporter	-3.21
SPD_1589	*SPD_1589*	Putative Hemin transporter	-3.26
SPD_1590	*SPD_1590*	Putative Hemin transporter	-2.69
SPD_1591	*SPD_1591*	Putative Hemin transporter	-2.50
SPD_1927	*ctpC*	Cation-transporting ATPase (EC 3.6.3.-)	-1.59

a. Log_2-fold change in gene expression comparing *S. pneumoniae* D39 in CDM supplemented with 30 μM Cd^{2+} treatment relative to CDM alone.
