# Supplementary material for: Cadmium stress dictates central carbon flux and alters membrane composition in Streptococcus pneumoniae
Source: Commun Biol. 2020 Nov 19;3:694. doi: 10.1038/s42003-020-01417-y (PMC7678824; doi:10.1038/s42003-020-01417-y)
Supplement: Supplementary file 2 — Description of Additional Supplementary Files [file 42003_2020_1417_MOESM2_ESM.pdf]

Description of Additional Supplementary Files

File Name: Supplementary Data 1
Description: **Proteins identified by mass spectrometry.** List of all proteins identified by mass spectrometry in each analysed anion exchange fraction. Only proteins that reached the significance threshold of 2 peptides hits and a score of >24 were considered true identifications.

File Name: Supplementary Data 2
Description: **Source data for main Figures.** Source data for all main text figures that do not have an associated data repository. Source data has been provided for Fig. 2a-c, Fig. 3, Fig. 5 and Fig. 6a,b.
