# Supplementary material for: Cadmium stress dictates central carbon flux and alters membrane composition in Streptococcus pneumoniae
Source: Commun Biol. 2020 Nov 19;3:694. doi: 10.1038/s42003-020-01417-y (PMC7678824; doi:10.1038/s42003-020-01417-y)
Supplement: Supplementary file 5 — Reporting Summary [file 42003_2020_1417_MOESM5_ESM.pdf]

nature research

Corresponding author(s): Stephanie Neville
Christopher McDevitt

Last updated by author(s): Oct 8, 2020

Reporting Summary

Nature Research wishes to improve the reproducibility of the work that we publish. This form provides structure for consistency and transparency in reporting. For further information on Nature Research policies, seeAuthors & Referees and theEditorial Policy Checklist .

Statistics

For all statistical analyses, confirm that the following items are present in the figure legend, table legend, main text, or Methods section.

n/a	Confirmed	
☐	☒	The exact sample size (*n*) for each experimental group/condition, given as a discrete number and unit of measurement
☐	☒	A statement on whether measurements were taken from distinct samples or whether the same sample was measured repeatedly
☐	☒	The statistical test(s) used AND whether they are one- or two-sided<br>*Only common tests should be described solely by name; describe more complex techniques in the Methods section.*
☒	☐	A description of all covariates tested
☐	☒	A description of any assumptions or corrections, such as tests of normality and adjustment for multiple comparisons
☐	☒	A full description of the statistical parameters including central tendency (e.g. means) or other basic estimates (e.g. regression coefficient) AND variation (e.g. standard deviation) or associated estimates of uncertainty (e.g. confidence intervals)
☐	☒	For null hypothesis testing, the test statistic (e.g. *F*, *t*, *r*) with confidence intervals, effect sizes, degrees of freedom and *P* value noted<br>*Give P values as exact values whenever suitable.*
☒	☐	For Bayesian analysis, information on the choice of priors and Markov chain Monte Carlo settings
☒	☐	For hierarchical and complex designs, identification of the appropriate level for tests and full reporting of outcomes
☒	☐	Estimates of effect sizes (e.g. Cohen's *d*, Pearson's *r*), indicating how they were calculated

Our web collection on statistics for biologists contains articles on many of the points above.

Software and code

Policy information about availability of computer code

Data collection	No software was used
Data analysis	All graphing and statistical analyses were conducted in GraphPad Prism 8 (commercially available). Protein Mass Spectrometry data was analysed using Matrix Scientific MASCOT MS/MS software (commercially available).

For manuscripts utilizing custom algorithms or software that are central to the research but not yet described in published literature, software must be made available to editors/reviewers. We strongly encourage code deposition in a community repository (e.g. GitHub). See the Nature Research guidelines for submitting code & software for further information.

Data

Policy information about availability of data

All manuscripts must include a data availability statement. This statement should provide the following information, where applicable:

- Accession codes, unique identifiers, or web links for publicly available datasets
- A list of figures that have associated raw data
- A description of any restrictions on data availability

RNA sequencing data have been deposited in NCBI Gene Expression Omnibus databank under submission identifier GSE141681. The mass spectrometry proteomics data have been deposited to the ProteomeXchange Consortium via the PRIDE partner repository with the dataset identifier PXD021643 and 10.6019/PXD021643. Raw data files for all other Figures are available from the corresponding authors upon reasonable request.

Field-specific reporting

Please select the one below that is the best fit for your research. If you are not sure, read the appropriate sections before making your selection.

Life sciences study design

All studies must disclose on these points even when the disclosure is negative.

Sample size	Sample sizes were derived based on the accepted conventions for life sciences research i.e. for independent biological replicates, n=3 is sufficient for performing statistical comparisons between experimental groups. For the -omics analyses, sample sizes were guided by industry standards i.e. RNA sequencing used 4 pooled biological replicates (Australian Genome Research Facility), metabolomic analyses used 6 biological replicates (Metabolon), metalloproteomic maps and mass spectrometry data were generated from single biological replicates to allow direct correlation between metal ion determination and protein identity.
Data exclusions	No data was excluded from the analyses.
Replication	Data reproducibility was ensured through temporal separation of replicate experiments. Experiments contributing to a single dataset were performed across multiple days/weeks to ensure that analyses were being conducted with multiple bacterial cultures, reagents etc. This ensures that all generated data are robust and reproducible. All attempts at reproducibility were successful.
Randomization	Randomization is not relevant for this study because whole bacterial cultures were analysed. Single colony analyses were not conducted.
Blinding	Blinding was not necessary for this study as group allocation and/or randomization was not applied.

Reporting for specific materials, systems and methods

We require information from authors about some types of materials, experimental systems and methods used in many studies. Here, indicate whether each material, system or method listed is relevant to your study. If you are not sure if a list item applies to your research, read the appropriate section before selecting a response.

Materials & experimental systems

n/a	Involved in the study
☒	☐ Antibodies
☒	☐ Eukaryotic cell lines
☒	☐ Palaeontology
☒	☐ Animals and other organisms
☒	☐ Human research participants
☒	☐ Clinical data

Methods

n/a	Involved in the study
☒	☐ ChIP-seq
☒	☐ Flow cytometry
☒	☐ MRI-based neuroimaging
